# Cytotoxic and Anti-Inflammatory Effects of *Ent*-Kaurane Derivatives Isolated from the Alpine Plant *Sideritis hyssopifolia*

**DOI:** 10.3390/molecules25030589

**Published:** 2020-01-29

**Authors:** Axelle Aimond, Kevin Calabro, Coralie Audoin, Elodie Olivier, Mélody Dutot, Pauline Buron, Patrice Rat, Olivier Laprévote, Soizic Prado, Emmanuel Roulland, Olivier P. Thomas, Grégory Genta-Jouve

**Affiliations:** 1 Laboratoire de Chimie-Toxicologie Analytique et Cellulaire (C-TAC) UMR CNRS 8038 CiTCoM Université Paris-Descartes, 4, Avenue de l’Observatoire, 75006 Paris, France; axelle.aimond@clarins.com (A.A.); elodie.eolivier@gmail.com (E.O.); melodydutot@gmail.com (M.D.); pauline.buron92@gmail.com (P.B.); patrice.rat@parisdescartes.fr (P.R.); olivier.laprevote@parisdescartes.fr (O.L.); 2 Laboratoires Clarins, 5 Rue Ampère, 95300 Pontoise, France; coralie.audoin@clarins.com; 3 Marine Biodiscovery, School of Chemistry and Ryan Institute, National University of Ireland Galway (NUI Galway), University Road, H91 TK33 Galway, Ireland; kevin.calabro@nuigalway.ie; 4 Muséum National d’Histoire Naturelle, Unité Molécules de Communication et Adaptation des Micro-Organismes, UMR 7245, CP 54, 57 rue Cuvier, 75005 Paris, France; sprado@mnhn.fr (S.P.); emmanuel.roulland@parisdescartes.fr (E.R.); 5 Laboratoire Ecologie, Evolution, Interactions des Systèmes Amazoniens (LEEISA), USR 3456, Université De Guyane, CNRS Guyane, 275 Route de Montabo, 97334 Cayenne, French Guiana

**Keywords:** *Sideritis hyssopifolia*, *ent*-kaurane, anti-inflammatory, NMR

## Abstract

This paper reports the isolation and structural characterization of four new *ent*-kaurane derivatives from the Lamiaceae plant *Sideritis hyssopifolia*. Planar structures and relative configurations were determined using both mass spectrometry and nuclear magnetic resonance (1D and 2D). Absolute configurations were determined by comparing experimental and theoretical electronic circular dichroism spectra. The cytotoxic and microbial activities of all new compounds were tested. Compounds that were non-cytotoxic were further evaluated for anti-inflammatory activity.

## 1. Introduction

**Figure 1:**
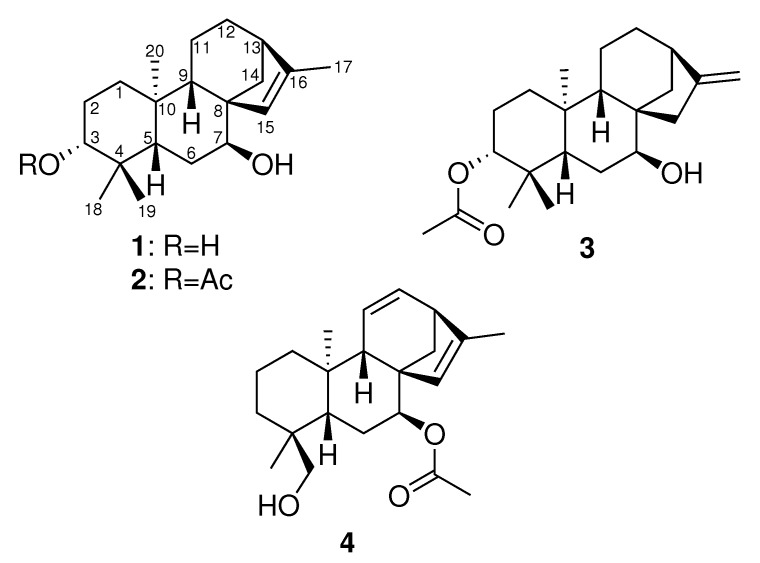
Structure of the new *ent*-kauranes compounds **1**–**4**.

The cosmetics sector represents a huge potential for growth in today’s society. The quest for wellness and beauty has led to an intensive search for new products that can improve both the appearance and hygiene of an aging population. In this context, an increasing number of cosmetic companies are using nature as a unique source for their formulations. To discover new compounds of interest, the French company Laboratoires Clarins has been conducting an in-depth investigation of plants in their unique outdoor fields situated in the French Alps. *Sideritis*, containing around 190 species, is a widespread genus of the family Lamiaceae, which is commonly found in the Northern Hemisphere [1]. Around 250 natural products have been reported from the genus *Sideritis*, of which 160 are in the diterpene class, specifically in the kaurane group [2,3]. These kauranes and their enantiomers, *ent*-kauranes, exhibit a wide range of bioactivities, with antioxidant, anti-tyrosinase, anti-cholinesterase, and anti-inflammatory properties reported [4,5,6]. The focus of this study was the species *Sideritis hyssopifolia* grown in the French Alps. The phytochemical composition of this species is poorly described, with only two publications reporting the isolation and characterization of its flavonoids and its essential oil composition [7,8]. Except for siderol [9], diterpenoids have yet to be reported in *S. hyssopifolia* [10]. Because these compounds are commonly found throughout the *Sideritis* genus, we re-investigated the phytochemical composition of *S. hyssopifolia* and, as a result, isolated 12 compounds, eight of which are reported in this species for the first time, namely, siderol [9], sideridiol [9], siderone [11], *ent*-kaurene I [12], sideritriol [13], *ent*-15β,16β-epoxykauran-18-ol [14], *epi*-candicandiol [12], and *ent*-3β-7α-dihydroxykaur-16-ene [15]. Additionally, four new *ent*-kauranes were fully characterized (Figure 1) using 1D and 2D NMR, MS, and ECD. The four novel compounds were evaluated for their cytotoxic, antimicrobial, and anti-inflammatory activities.

## 2. Results and Discussion

**Figure 2:**
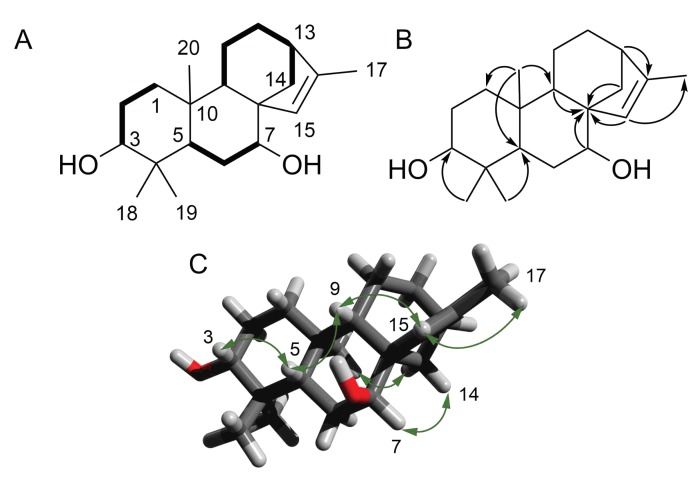
(**A**) COSY correlations; (**B**) HMBC key correlations; and (**C**) NOESY correlations.

Compound **1** was isolated as a light yellow oil. Its molecular formula was determined from the [M+H]${}^{+}$ observed at *m*/*z* 305.2481 (calculated for $C_{20}H_{33}O_{2}$, 305.2475, δ 1.9 ppm) in the HRESIMS spectrum acquired in positive ionization mode; the formula required five degrees of unsaturation in the molecule. The ${}^{1}$H NMR spectrum exhibited four methyl singlets at δ 0.76 (s, 3H, H${}_{3}$-18), 0.95 (s, 3H, H${}_{3}$-19), 1.07 (s, 3H, H${}_{3}$-20), and 1.71 (s, 3H, H${}_{3}$-17) ppm (Table 1 and Table 2). A signal corresponding to a trisubstituted double bond at δ 5.52 (s, 1H, H-15) was also observed, together with two hydroxylated methines at δ 3.17 (dd, *J* = 11.6, 4.5 Hz, 1H, H-3) and 3.54 (br s, 1H, H-7). Inspection of the ${}^{1}$H–${}^{1}$H COSY spectrum led to the identification of three spin systems. The first one started with a clear correlation of H-3 with H-2a δ 1.67 (m, 1H) and H-2b δ 1.60 (m, 1H), which, in turn, correlated with the methylene protons H-1 at δ 1.84 (1H, m) and 0.95 (1H, m). A second spin system was observed between H-7 and H-6 δ 1.66 (m, 1H) and 1.62 (m, 1H), and the last correlation was observed between H6b and H-5 at δ 1.40 (d, *J* = 12.0 Hz, 1H). A last spin system comprising H-9/H-11/H-12/H-13/H-14 was identified, with correlations between δ 1.28 (d, *J* = 4.7 Hz, 1H, H-9), 1.54 (m, 2H, H${}_{2}$-11), 1.51 (m, 2H, H${}_{2}$-12), 2.33 (br s, 1H, H-13), and 1.96 (d, *J* = 9.9 Hz, 1H, H-14a). A ${}^{4}$*J* cross-peak between H-15 and H-17 was also observed. Analysis of the HMBC spectrum revealed a connection between the three spin systems. The correlations of H-20 with C-1/C-5/C-9 and H-18/H-19 with C-3/C-5 indicated the presence of the first decalin ring. Two other cycles were identified using intense ${}^{2}$*J* correlations between H-7/H-9/H-14/H-15 and C-8. The last cycle was confirmed by the cross-peak between H-13 and C-16. The full three-dimensional structure was determined using the coupling constant values of the ${}^{1}$H NMR spectra and the NOESY correlations (see Figure 2).

The multiplicity of the signal at δ 3.17 (dd, *J* = 11.6, 4.5 Hz, 1H, H-3) indicated an axial orientation for H-3. The NOESY correlations between H-5/H-3 and H-5/H-9 confirmed the relative configuration of C-5 and the trans ring junction. The position of the trisubstituted double bond was supported by the NOESY correlation between H-15/H-9 and the correlation between H-14/H-20. Finally, the broad singlet signal for H-7 indicated a α orientation at C-7. This was further confirmed by the correlation between H-7/H-14b.

**Figure 3:**
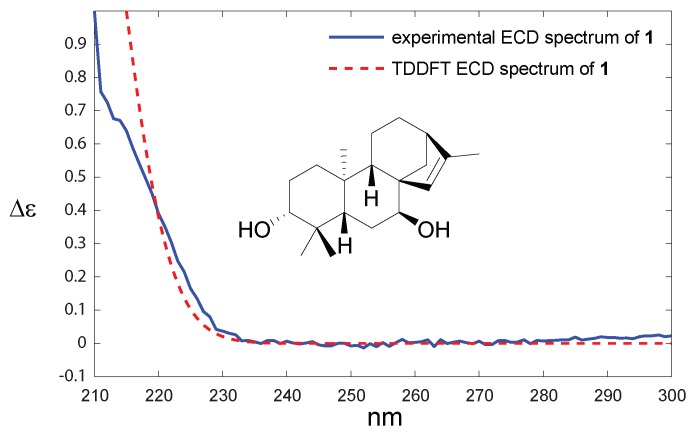
Comparison between experimental and theoretical ECD spectra of compound **1**.

The absolute configuration was determined by comparing experimental and theoretical electronic circular dichroism (ECD) spectra. As expected, the spectrum (Figure 3) revealed only one Cotton Effect (CE), which was due to the $\pi \to \pi^{*}$ transition. The sign of the CE agreed with compound **1** belonging to the *ent*-kaurane series. This compound was named hyssopifoliol A.

Compound **2** was isolated as a light yellow oil. Its molecular formula was determined from the [M-AcOH+H]${}^{+}$ observed at *m*/*z* 287.2361 (calculated for $C_{20}H_{31}O$, 287.2369, Δ−2.8 ppm) in the HRESIMS spectrum acquired in positive ionization mode. Inspection of the ${}^{1}$H NMR spectrum suggested that compound **2** was very similar to compound **1** since only one additional signal, which was easily assigned to an acetyl moiety, was found at δ 2.04 (s, 3H, H${}_{3}$-22) ppm. This was confirmed by the deshielding of the H-3 signal at δ 4.52 (dd, *J* = 11.6, 4.6 Hz, 1H, H-3) and the HMBC correlation between H-3 and C-21. The three-dimensional structure appeared to be similar to that determined for compound **1** because the coupling constants observed for the critical signals did not change. The multiplicities were similar with a doublet of doublets at δ 4.52 (dd, *J* = 11.6, 4.5 Hz) and 3.63 (*J* = 2.9 Hz) ppm for H-3 and H-7, respectively. The relative configurations at C-5, C-8, and C-9 were established using the correlations in the NOESY spectrum (see Supporting Information). The absolute configuration of compound **2** was determined by comparing the sign of the unique CE on the ECD spectrum. As for compound **1**, the positive band at ca. 215 nm led to the conclusion that compound **2** was an *ent*-kaurane and was named hyssopifoliol B.

Compound **3** was isolated as a light yellow oil. Its molecular formula was determined from the [M+H]${}^{+}$ observed at *m*/*z* 347.2786 (calculated for $C_{22}H_{34}O_{3}Na$, 347.2780, δ +1.7 ppm) in the HRESIMS spectrum acquired in positive ionization mode; the formula required 6 degrees of unsaturation in the molecule. The ${}^{1}$H NMR spectrum exhibited four methyl singlets at δ 0.86 (s, H${}_{3}$-18), 0.86 (s, H${}_{3}$-19), 1.05 (s, 3H, H${}_{3}$-20), and 2.04 (s, 3H, H${}_{3}$-22) ppm. The signal at 4.49 (dd, *J* = 11.4, 5.2 Hz) is typical of an axial acetylated methine at H-3, and a broad singlet assigned to a hydroxylated methine at H-7 was observed at δ 3.62 (t, *J* = 3.1 Hz, 1H, H-7). A major difference was observed between compounds **2** and **3**: two broad one-proton singlets at δ 4.76 and 4.80 ppm were observed in the ${}^{1}$H NMR spectrum, while the singlet at δ 1.71 was absent. This difference was attributed to the isomerization of the double bond to obtain an *exo*-methylene group (H${}_{2}$-17). The relative configuration was conserved between compounds **2** and **3**, and the multiplicities of the key stereogenic centers were very similar for both compounds (see Table 1). The relative configuration of 3 was confirmed by saponification $\left(K_{2}CO_{3}/MeOH\right)$. The ${}^{1}$H NMR spectrum of the resulting diol compound was compared with the published spectrum of *ent*-3β-7α-dihydroxykaur-16-ene [15], and a perfect match was obtained, thus confirming the relative configuration of compound **3**. According to the sign of the CE on ECD spectrum, the same absolute configuration was attributed to compound **3**. Compound **3** was thus named 3-acetoxy-*ent*-3β-7α-dihydroxykaur-16-ene.

Compound **4** was isolated as a light yellow oil. Its molecular formula was determined from the [M-AcOH+H]${}^{+}$ observed at *m*/*z* 285.2251 (calculated for $C_{20}H_{29}O$, 285.2221, δ 10.5 ppm) in the HRESIMS spectrum acquired in positive ionization mode; the formula required seven degrees of unsaturation in the molecule. This additional unsaturation was quickly identified in the ${}^{1}$H NMR spectrum as two olefinic signals at δ 5.36 (dd, *J* = 9.7, 3.5 Hz, 1H, H-11) and 6.25 (dd, *J* = 9.6, 6.5 Hz, H-12). The core of compounds **1** and **2** was conserved, but a methyl singlet was missing, and two oxygenated geminal protons were observed at δ 3.36 (d, *J* = 10.8 Hz, H-19a) and 3.04 (d, *J* = 10.8 Hz, 1H, H-19b). Oxidation at C-19 was confirmed by the HMBC correlations between H-19 and C-3/C-4/C-5/C-18. The acetyl group was positioned at C-7, as determined by the deshielding of H-7. This was further confirmed by the cross-peak at H-7/C-21 in the HMBC spectrum. Compound **4** was named 11,12-didehydrosiderol.

**Figure 4:**
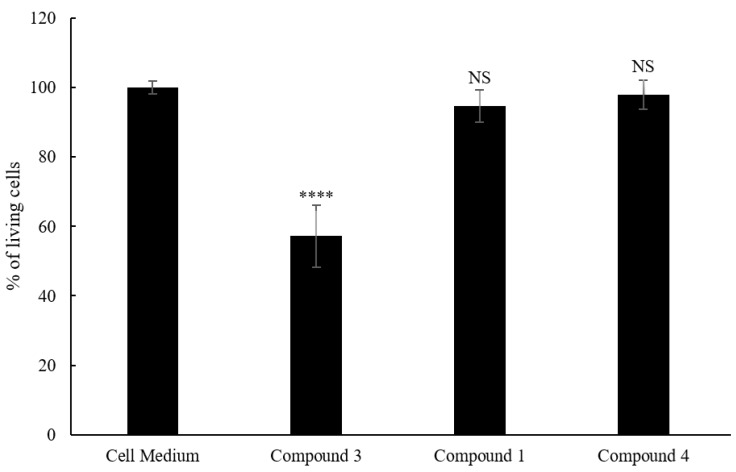
Viability of HaCaT cells after 6 h of incubation with compounds **1**, **3** and **4**. (NS: not significant; ****: *p* < 0.0001).

After an evaluation of the purity of the compounds, compounds **1** (97%), **3** (95%) and **4** (87%) were tested for cytotoxicity and activity against *Staphylococcus aureus* (ATCC 6538). Unfortunately, no antimicrobial activity was detected at the tested concentrations (data not shown). Compound **2** was indeed not tested for biological activity due to its low purity (<75%). Additionally, as shown in Figure 4, compound **3** was found to be cytotoxic to HaCaT cells at a concentration of 10 μg/mL (cell viability was reduced to ca. 60%), so it was excluded from subsequent testing for anti-inflammatory activity. It was previously reported that the exomethylene cyclopentanone moiety is beneficial for anticancer activity on cancer cell lines [16]. The *exo*-methylene group present in compound **3** and absent in compounds **1** and **4** may be therefore responsible for the cytotoxicity on the spontaneously immortalized HaCaT cell line.

**Figure 5:**
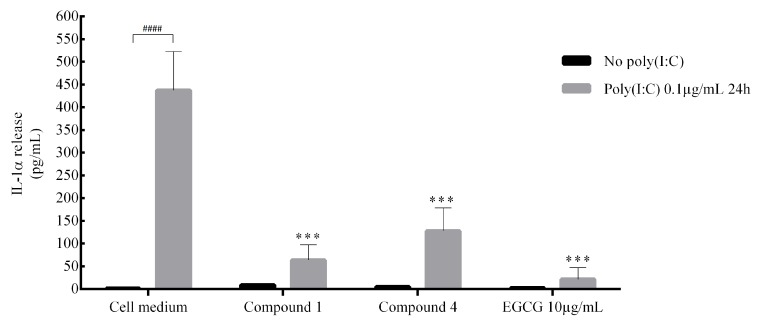
Anti-inflammatory activity of compounds **1** and **4** in HaCaT cells stimulated by poly(I:C). Compounds in ethanol were prepared at 1:100 dilution in cell medium to obtain a concentration of 10 μg/mL (***: *p* < 0.001 compared to cell medium with poly(I:C); ####: *p* < 0.0001).

Compounds **1** and **4** were tested for potential anti-inflammatory activity by incubating human keratinocytes with the compounds for 24 hours, followed by stimulation of the cells with poly(I:C). As shown in Figure 5, poly(I:C) induced a marked elevation in the release of IL-1α (250-fold). Preincubation with compounds **1** and **4** significantly inhibited the release of IL-1α. These results demonstrate that two of the new diterpenoids extracted from *S. hyssopifolia* have anti-IL-1α effects on skin epithelial cells. Human keratinocytes constitutively synthesize proIL-1α and -β but they do not activate and secrete these proinflammatory cytokines under normal conditions [17]. Upon activation, IL-1 signalling is involved in many (auto)-inflammatory skin diseases like psoriasis [18], vitiligo [19] or melanoma [20]. The new diterpenoids extracted from *S. hyssopifolia* represent new therapeutic topical agents via the modulation of IL-1α secretion.

## 3. Materials and Methods

### 3.1. General Experimental Procedures

Optical rotation was measured at the Na D-line (589.3 nm) with a 5 cm cell at 20 °C on a UniPol L1000 polarimeter (Schmidt + Haensch, Berlin, Germany) in methanol. UV and ECD data were obtained using a ChirascanTM V100 (Applied Photophysics, Leatherhead, UK) in acetonitrile. NMR experiments were performed using an Inova 500 MHz spectrometer (Varian, Palo Alto, CA, USA). Chemical shifts were referenced in ppm to the residual solvent signals (CD${}_{3}$OD, at $\delta_{H}$ 3.31 and $\delta_{C}$ 49.00 ppm; CDCl${}_{3}$, at $\delta_{H}$ 7.26 and $\delta_{H}$ 77.0 ppm). High-resolution mass spectra were obtained using a mass spectrometer (HRMS; Agilent 6540, Santa Clara, CA, USA). Preparative liquid chromatography was performed using a Jasco system (Tokyo, Japan) equipped with a PU-2087 pump and a UV-2075 detector, while semi-preparative purification was realized using a Waters 2690 system (Milford, MA, USA) equipped with the UV detector 2487.

### 3.2. Plant Material

Flowering tops of *S. hyssopifolia* were collected in the Alps, France, at an altitude of 1300 m. Botanical identification of the species was carried out by an ethnobotanist of Laboratoires Clarins. The samples were protected from direct light and air-dried at room temperature for three days. Two harvests were carried out (July 2016 and August 2017). After comparing the phytochemical composition, the harvests were gathered.

### 3.3. Extraction and Isolation

Dried powder of the aerial parts of *S. hyssopifolia* (300 g) was successively extracted three times with a methanol/dichloromethane mixture (1:1, *v*/*v*) for 15 min under sonication at room temperature. The crude extract (27 g) was further fractionated by reversed-phase (C${}_{18}$) vacuum liquid chromatography (VLC) using a step gradient of H${}_{2}$O/MeOH (100:0, 75:25, 50:50, 25:75, 0:100) followed by a step gradient of MeOH/DCM (50:50, 0:100) to yield seven fractions (F1: 4.56 g, F2: 3.67 g, F3: 2.76 g, F4: 5.93 g, F5: 5.47 g, F6: 3.33 g, and F7: 0.64 g). The methanolic fraction (F5, m = 860 mg) was purified by preparative reversed-phase high-performance liquid chromatography with a mixture of water and acetonitrile (flow: 12 mL/min; solvent A: H${}_{2}$O; solvent B: acetonitrile; gradient: 0–3 min 72% B, 3–15 min 72 → 86% B, 15–20 min 86% B, 20–21 min 86% → 72% B, 21–25 min 72% B) to obtain 12 peaks (P1→P12). After ${}^{1}$H NMR and MS analyses, only compound **1** was pure enough to be identified (6.43 mg, *t*${}_{R}$ = 11 min). The other compounds were identified after a second purification of peaks 4, 7, and 8 (see Supporting Information). Peak 4 (37 mg, 50 mg/mL) was subjected to preparative HPLC (column A, flow: 3.5 mL/min; solvent A: H${}_{2}$O; solvent B: acetonitrile, isocratic 55% B, 31 min) to obtain three known molecules and compound **2** (2.5 mg, *t*${}_{R}$ = 11.6 min). Peak 7 (3.8 mg, 5 mg/mL) was subjected to semi-preparative HPLC (system: B, column B, flow: 1 mL/min; solvent A: H${}_{2}$O; solvent B: acetonitrile, isocratic 47% B, 43 min) to obtain a known molecule and compound **3** (0.99 mg, *t*${}_{R}$ = 33.5 min). Peak 8 (7.5 mg, 5 mg/mL) was subjected to semi-preparative HPLC (system: B, column B, flow: 1 mL/min; solvent A: H${}_{2}$O; solvent B: acetonitrile, isocratic 49% B, 40 min) to yield compound **4** (0.53 mg, *t*${}_{R}$ = 34 min).

### 3.4. Computational Details

All QM calculations were carried out using Gaussian 16. The GMMX package was used for the conformational analysis (force field: MMFF94). TD DFT calculations were performed using the B3LYP method at the 6-31G(d) level for 20 excited states. GaussView 6.0 was used to plot the ECD spectra.

### 3.5. Cell Experiments

HaCaT cells (spontaneously transformed human keratinocytes) were obtained from Cell Lines Service (CLS; Eppelheim, Germany). The cells were cultured in Dulbecco’s Modified Eagle Medium (DMEM; Gibco, France) supplemented with 10% fetal bovine serum, 2 mM glutamine, 50 IU/mL penicillin, and 50 IU/mL streptomycin (Gibco) at 5% CO${}_{2}$ and 37 °C. When the HaCaT cells reached confluency, they were dispersed using trypsin and counted using a hematimeter. The cell suspension was diluted to a cell density of 100,000 cells/mL and seeded in 96-well microplates for 24 h. The compounds in ethanol were prepared at 1:100 dilution in culture medium and incubated for 24 h. Epigallocatechin gallate (EGCG) at 10 µg/mL was used as an anti-inflammatory control [21].

### 3.6. Cell Viability Evaluation

A cell viability assay was carried out using the resazurin salt assay. Briefly, after incubating the cells with compounds **1**–**4**, the medium was removed, and 200 μL of resazurin solution (9 mg/mL, m/v, Alfa Aesar by Thermo Fisher Scientific, Kandel, Germany) prepared in fresh medium was added to each well. Following 6 h of incubation, resorufin fluorescence was quantified at the respective excitation and emission wavelengths of 535 and 600 nm using a Tecan Spark microplate reader. The percentage of cell viability is expressed in relative fluorescence units (RFU) compared with the control cells (RFU × 100).

### 3.7. Interleukin Release Measurement

The potential anti-inflammatory effects of the compounds were evaluated by inducing cytokine release with poly(I:C), a synthetic analog of viral dsRNA (Sigma-Aldrich, Lyon, France) at 1 μg/mL for 24 hours in culture medium. The control group was incubated with culture medium. IL-1α release was quantified in supernatants by ELISA (DuoSet ELISA, R&D Systems, Minneapolis, MN) according to the manufacturer’s instructions. The absorbance signal was read at 450 nm using a microplate reader (Spark).

### 3.8. Saponification of Compound **3**

$K_{2}CO_{3}$ (50 mg, 0.36 mmol) was added to a methanol solution (1 mL) of compound **3** (0.53 mg, 0.0015 mmol) at room temperature. The reaction was monitored by TLC and quenched by pouring the reaction medium into brine. After extraction by ethylacetate, drying over anhydrous sodium sulfate, concentration under vacuum, and HPLC purification (heptane/ethylacetate, 1:1, *v*/*v*), the known compound *ent*-3β-7α-dihydroxykaur-16-ene (0.46 mg, 100%) was obtained. The spectral data of synthetic *ent*-3β-7α-dihydroxykaur-16-ene were identical to those of the natural compound [15].

### 3.9. Statistical Analyses

All statistical analyses were performed using R 3.5.0. Cell samples were analyzed by repeated measures (n = 3) one-way analysis of variance (ANOVA) followed by Dunnett’s test. Significant differences for compounds **1**–**4** are relative to the control and indicated in the results (NS: not significant; ***: *p* < 0.001; ****: *p* < 0.0001).

### 3.10. Hyssopifoliol A (Compound **1**)

Light yellow oil; [α]${}_{D}^{20}$−35 (*c* 0.1, MeOH); CD (acetonitrile) $\lambda_{max}$ (Δϵ) 210 (+0.11) nm; ${}^{1}$H and ${}^{13}$C NMR: see Table 1 and Table 2; MS: (+)-HRESIMS *m*/*z* 305.2481 (calcd. for $C_{20}H_{33}O_{2}$, 305.2475, Δ +1.9 ppm).

### 3.11. Hyssopifoliol B (Compound **2**)

Light yellow oil; [α]${}_{D}^{20}$−26 (*c* 0.07, MeOH); CD (acetonitrile) $\lambda_{max}$ (Δϵ) 210 (+0.06) nm; ${}^{1}$H and ${}^{13}$C NMR: see Table 1 and Table 2; MS: (+)-HRESIMS *m*/*z* 287.2361 (calcd. for $C_{20}H_{31}O$, 287.2369, Δ−2.8 ppm).

### 3.12. 3-Acetoxy-*ent*-3β-7α-dihydroxykaur-16-ene (Compound **3**)

Light yellow oil; [α]${}_{D}^{20}$−18 (*c* 0.06, MeOH); CD (acetonitrile) $\lambda_{max}$ (Δϵ) 210 (+0.05) nm; ${}^{1}$H and ${}^{13}$C NMR: see Table 1 and Table 2; MS: (+)-HRESIMS *m*/*z* 347.2786 (calcd. for $C_{22}H_{34}O_{3}Na$, 347.2780, Δ +1.7 ppm).

### 3.13. 11,12-Didehydrosiderol (Compound **4**)

Light yellow oil; [α]${}_{D}^{20}$−23 (*c* 0.08, MeOH); CD (acetonitrile) $\lambda_{max}$ (Δϵ) 215 (+0.07) nm; ${}^{1}$H and ${}^{13}$C NMR: see Table 1 and Table 2; MS: (+)-HRESIMS *m*/*z* 285.2251 (calcd. for $C_{20}H_{29}O$, 285.2221, Δ +10.5 ppm).

## 4. Conclusions

The chemical study of *S. hyssopifolia* led to the isolation and identification of several diterpenoid compounds. Eight compounds are reported for the first time in this species, and four are new *ent*-kauranes, which are designated hyssopifoliol A, hyssopifoliol B, 3-acetoxy-*ent*-3β-7α-dihydroxykaur-16-ene, and 11,12-didehydrosiderol. Biological tests showed that two of these molecules have good anti-inflammatory properties. Further biological tests will be performed to find potential cosmetics applications.

## Figures and Tables

**Table 1:** ${}^{1}$H NMR data of compounds **1**–**4** (${}^{1}$H 500 MHz).

	1 ${}^{a}$	2 ${}^{b}$	3 ${}^{b}$	4 ${}^{b}$
No.	$\delta_{H}$ (m, *J* in Hz)	$\delta_{H}$ (m, *J* in Hz)	$\delta_{H}$ (m, *J* in Hz)	$\delta_{H}$ (m, *J* in Hz)
1a	1.84 (dt, 13.0, 3.5)	1.82 (dt, 13.2, 3.5)	1.84 (m)	1.84 (m)
1b	0.95 (m)	1.03 (m)	1.05 (td, 13.0, 4.0)	1.07 (dd, 12.5, 3.6)
2a	1.67 (m)	1.70 (m)	1.69 (m)	1.65 (m)
2b	1.60 (m)	1.62 (m)	1.64 (m)	1.52 (m)
3a	3.17 (dd, 11.6, 4.5)	4.52 (dd, 11.6, 5.1)	4.53 (dd, 11.6, 5.0)	1.55 (m)
3b	-	-	-	1.27 (dd, 14.3, 5.5)
4	-	-	-	-
5	1.40 (d, 12.0)	1.49 (m)	1.55 (m)	1.86 (m)
6a	1.66 (m)	1.65 (m)	1.68 (m)	1.74 (dd, 14.4, 3.8)
6b	1.62 (m)			1.56 (m)
7	3.54 (bs)	3.63 (t, 2.9)	3.62 (t, 3.1)	4.82 (t, 3.0)
8	-	-	-	-
9	1.28 (d, 4.7)	1.30 (d, 7.5)	1.42 (d, 6.6)	1.86 (m)
10	-	-	-	-
11	1.54 (m)	1.49 (m)	1.56 (m)	5.36 (dd, 9.7, 3.5)
12a	1.51 (m)	1.49 (m)	1.70 (m)	6.25 (dd, 9.6, 6.5)
12b			1.49 (m)	-
13	2.33 (bs)	2.37 (bs)	2.68 (m)	2.56 (m)
14a	1.96 (d, 9.9)	1.89 d (10.1)	1.82 (m)	2.01 (d, 9.4)
14b	1.35 (dd, 10.1, 5.3)	1.36 (d, 10.2, 5.2)	1.17 (dd, 11.4, 5.0)	1.50 (m)
15	5.52 (s)	5.47 (s)	2.25 (bs)	5.09 (s)
16	-	-	-	-
17a	1.71 (s)	1.72 (bs)	4.83 (bs)	1.77 (d, 1.1)
17b	-	-	4.80 (bs)	-
18	0.96 (s)	0.84 (s)	0.88 (s)	0.72 (s)
19a	0.76 (s)	0.85 (s)	0.86 (s)	3.36 (d, 10.8)
19b	-	-	-	3.04 (d, 10.8)
20	1.07 (s)	1.05 (s)	1.05 (s)	1.04 (s)
21	-	-	-	-
22	-	2.04 (s)	2.04 (s)	2.09 (s)

${}^{a}$ CD${}_{3}$OD, ${}^{b}$ CDCl${}_{3}$.

**Table 2:** ${}^{13}$C NMR data of compounds **1**–**4** (${}^{13}$C 125 MHz).

	1 ${}^{a}$	2 ${}^{b}$	3 ${}^{b}$	4 ${}^{b}$
No.	$\delta_{C}$	$\delta_{C}$	$\delta_{C}$	$\delta_{C}$
1	40.1	38.4	38.4	39.2
2	28.1	23.6	23.8	17.9
3	79.8	80.8	81.0	35.1
4	39.4	37.2	37.4	37.1
5	46.1	45.1	45.6	38.8
6	27.7	26.7	27.4	23.9
7	76.2	75.0	77.2	78.0
8	54.5	53.3	48.3	50.5
9	45.2	43.7	50.3	50.9
10	40.3	39.1	38.9	38.8
11	19.6	18.4	18.0	126.1
12	25.9	24.8	33.7	134.4
13	46.1	44.7	43.9	45.5
14	43.5	42.1	38.6	40.2
15	131.9	129.7	45.8	126.6
16	144.2	144.3	154.9	154.0
17	15.5	15.5	103.8	15.9
18	162	16.6	16.9	17.6
19	28.6	28.0	28.3	71.3
20	18.2	17.6	17.6	17.5
21	-	170.9	171.1	170.5
22	-	21.3	21.5	21.5

${}^{a}$ CD${}_{3}$OD, ${}^{b}$ CDCl${}_{3}$.

## Supplementary Materials

The Supplementary Materials are available online.
