# Supplementary material for: Cytotoxic and Anti-Inflammatory Effects of Ent-Kaurane Derivatives Isolated from the Alpine Plant Sideritis hyssopifolia
Source: Molecules. 2020 Jan 29;25(3):589. doi: 10.3390/molecules25030589 (PMC7037520; doi:10.3390/molecules25030589)
Supplement: Supplementary file 1 [file molecules-25-00589-s001.pdf]

Cytotoxic and anti-inflammatory effects of *ent*-kaurane derivatives isolated from the Alpine plant *Sideritis hyssopifolia*

Axelle Aimond [1,2,†], Kevin Calabro [3,†], Coralie Audoin [2], Elodie Olivier [1], Mélody Dutot [1], Pauline Buron [1], Patrice Rat[1], Olivier Laprévote[1], Soizic Prado [4], Emmanuel Roulland [4], Olivier P. Thomas [3,*], and Grégory Genta-Jouve [1,5,*]

1 Laboratoire de Chimie-Toxicologie Analytique et Cellulaire (C-TAC) UMR CNRS 8038 CiTCoM Université Paris-Descartes, 4, avenue de l'Observatoire, 75006 Paris, France; gregory.genta-jouve@parisdescartes.fr (G.G.J.); olivier.laprevote@parisdescartes.fr (O.L.); Emmanuel Roulland (E.R)

2 Laboratoires Clarins, 5 rue Ampère, 95300 Pontoise, France; axelle.aimond@clarins.com (A.A.); coralie.audoin@clarins.com (C.A.)

3 Marine Biodiscovery, School of Chemistry and Ryan Institute, National University of Ireland Galway (NUI Galway), University Road, H91 TK33, Galway, Ireland; kevin.calabro@nuigalway.ie (K.C.); olivier.thomas@nuigalway.ie (O.P.T.)

4 Muséum National d'Histoire Naturelle, Unité Molécules de Communication et Adaptation des Micro-organismes, UMR 7245, CP 54, 57 rue Cuvier, 75005 Paris, France; sprado@mnhn.fr (S.P.)

5 Laboratoire Ecologie, Evolution, Interactions des Systèmes Amazoniens (LEEISA), USR 3456, Université De Guyane, CNRS Guyane, 275 Route de Montabo, 97334 Cayenne, French Guiana; gregory.genta-jouve@parisdescartes.fr (G.G.J.);

* Correspondence: olivier.thomas@nuigalway.ie (O.P.T.) gregory.genta-jouve@parisdescartes.fr (G.G.J.); Tel.: +353-9149-3563 (O.P.T.); +33153731585 (G.G.J.)

† These authors contributed equally to this work.

^{1}H NMR spectrum of **1** in CD_3OD (500MHz)

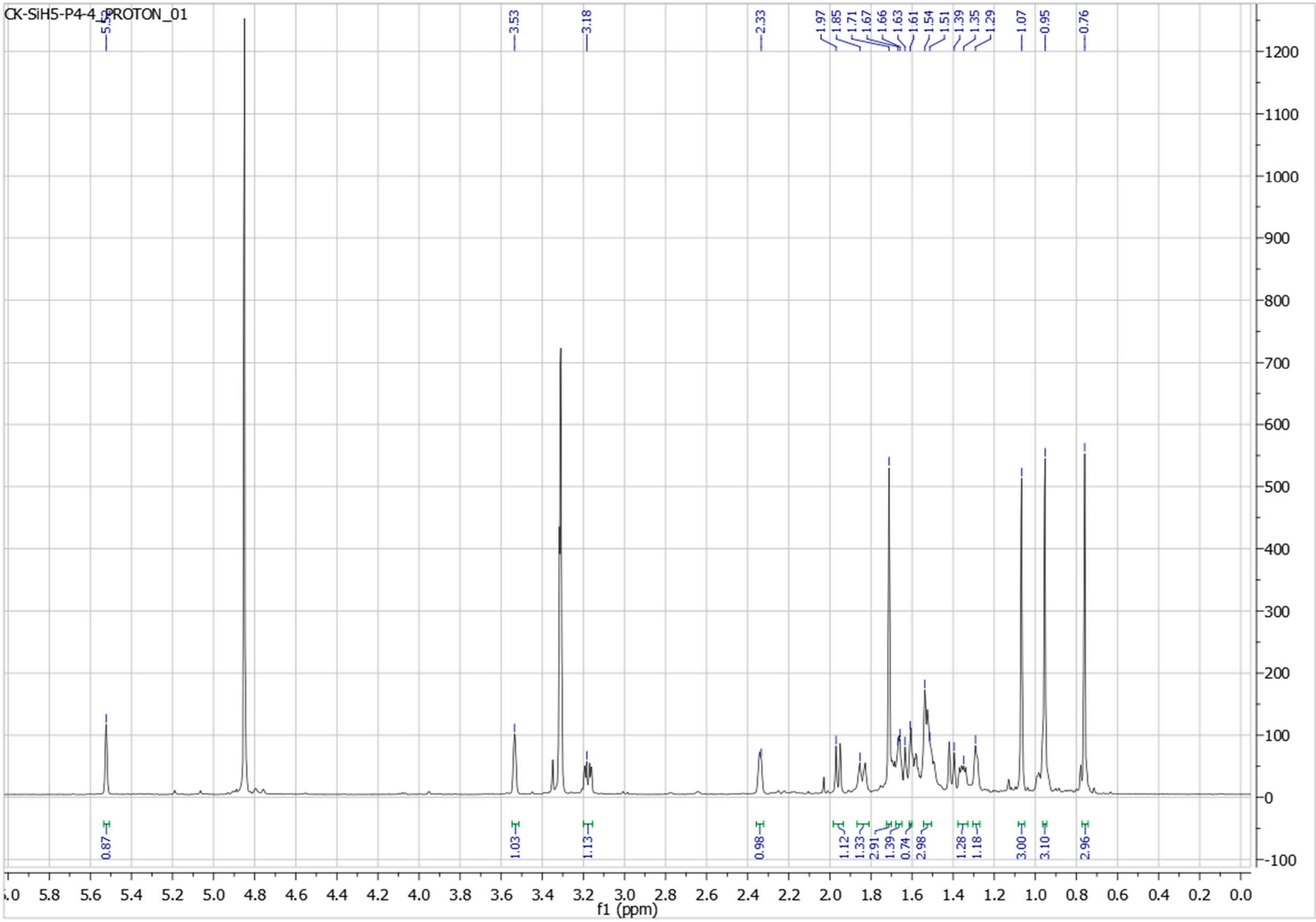

^{13}C NMR spectrum of **1** in CD_3OD (125MHz)

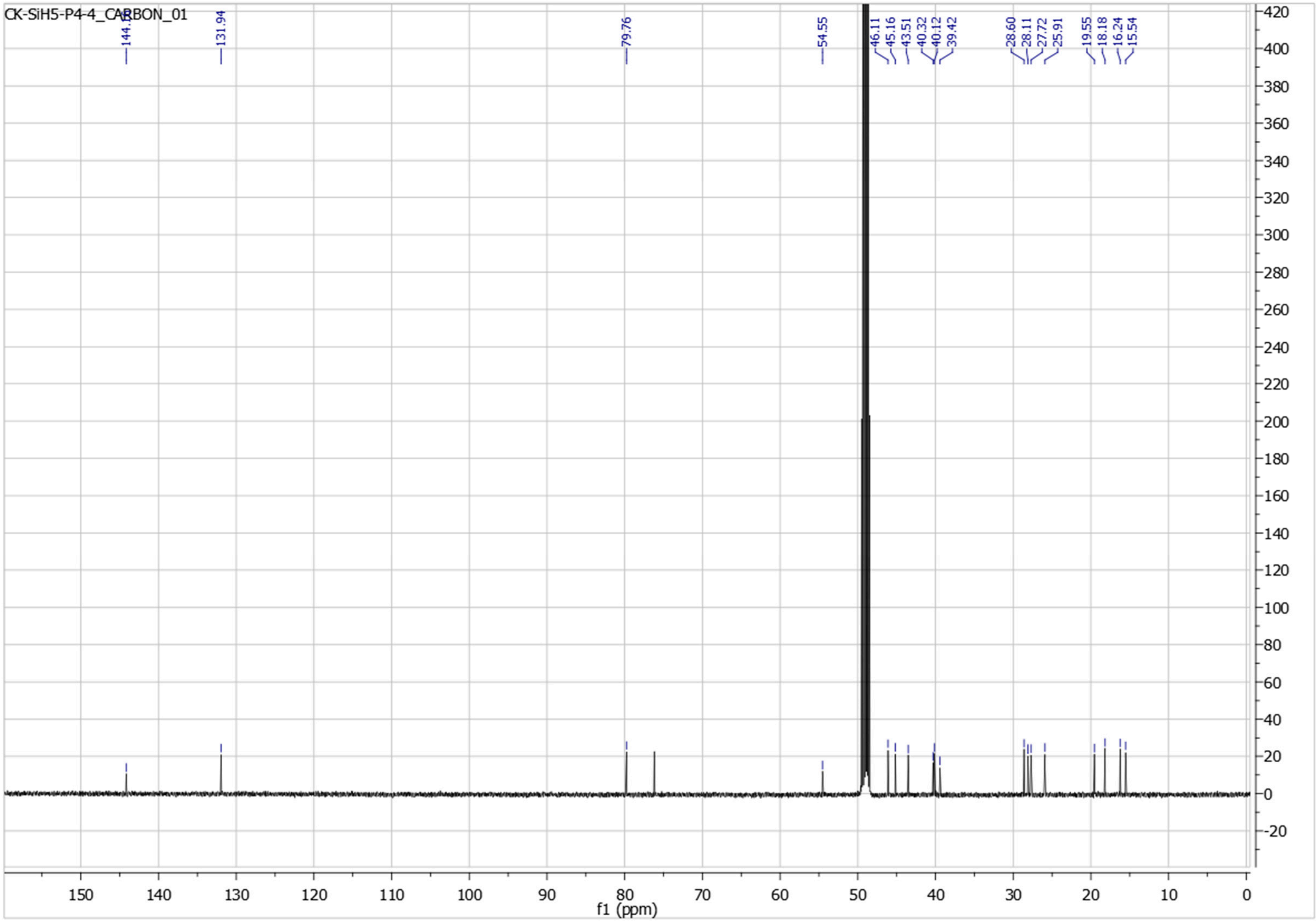

COSY NMR spectrum of **1** in CD_3OD (500MHz)

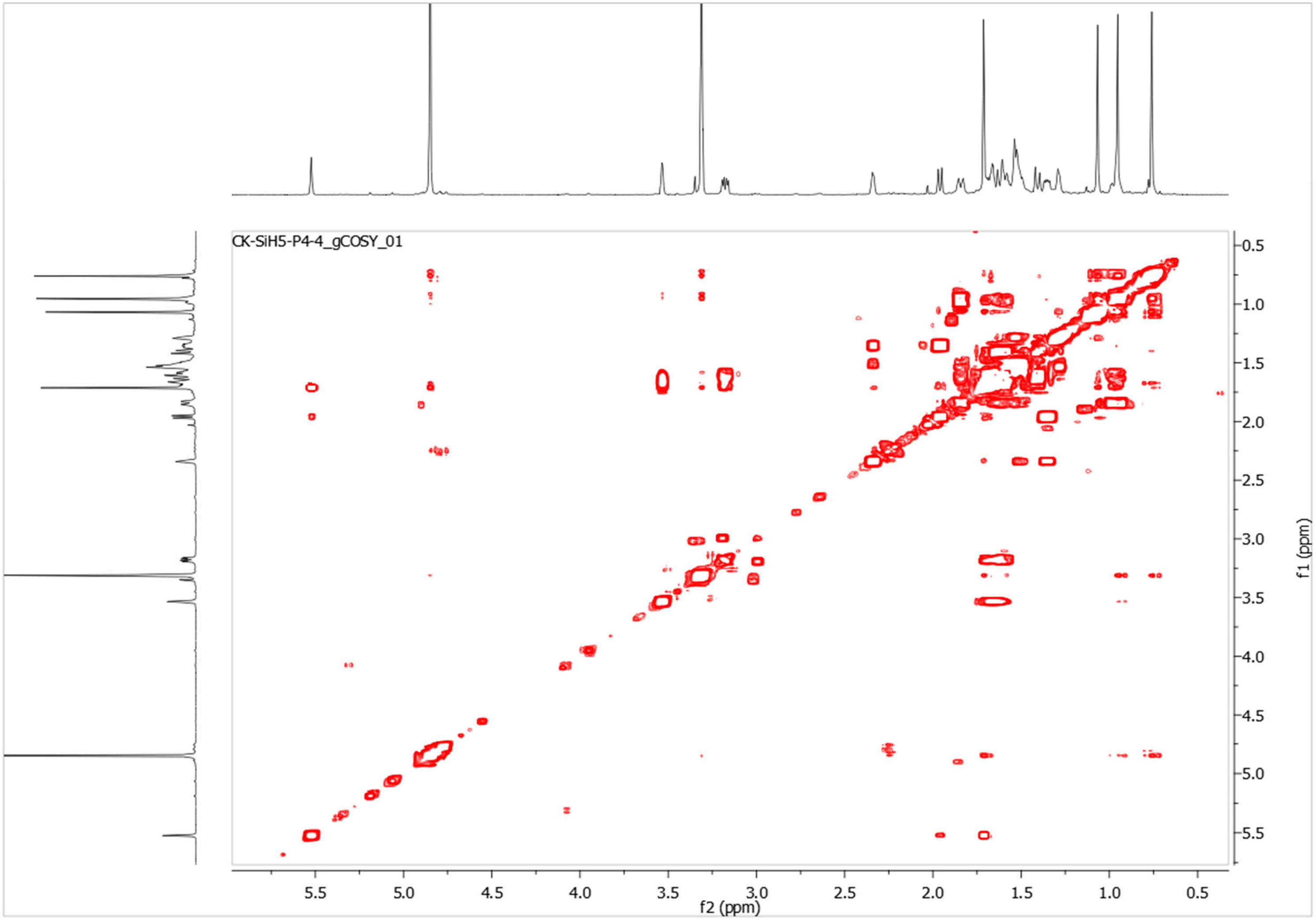

HSQC NMR spectrum of **1** in CD_3OD (500MHz)

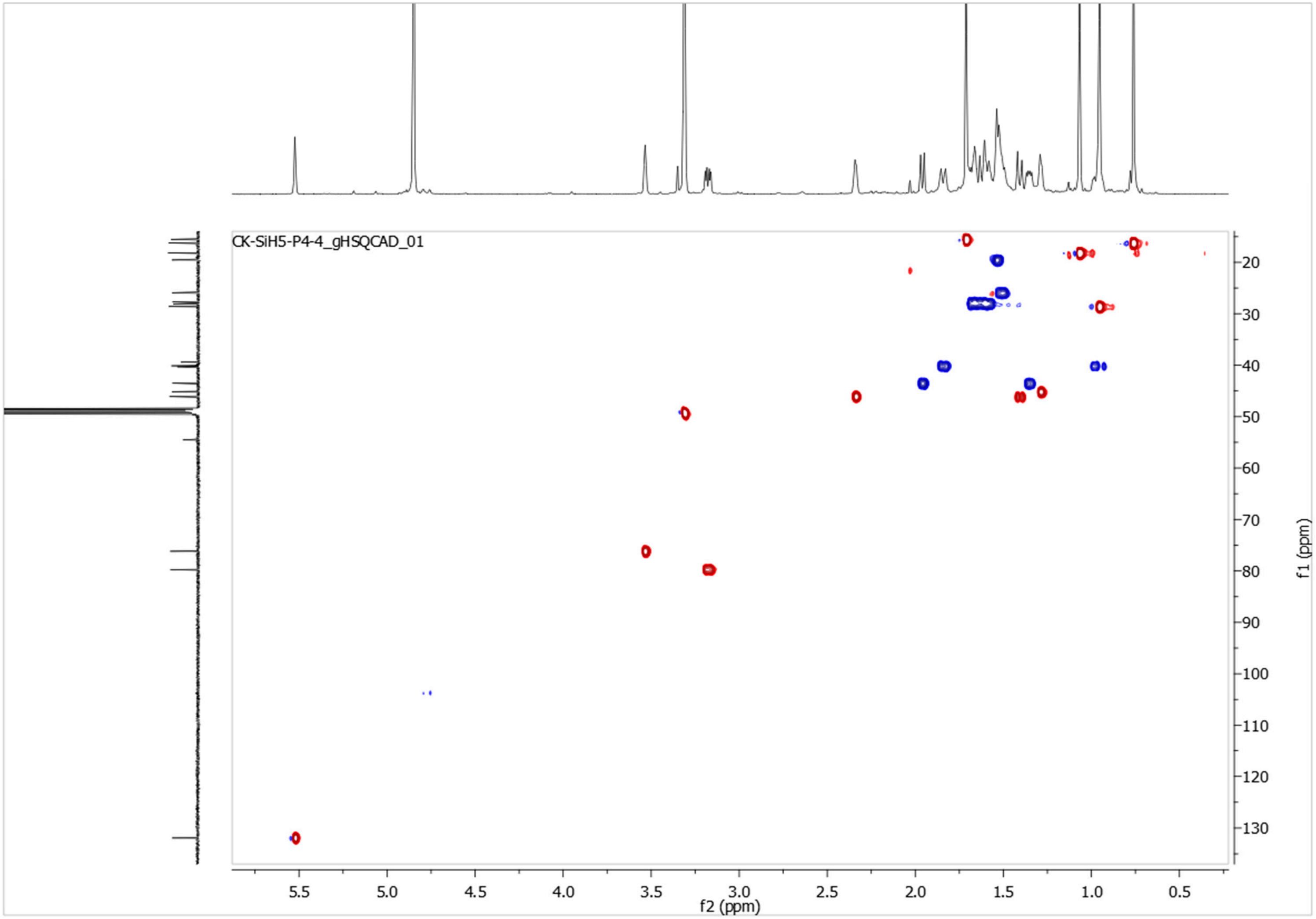

HMBC NMR spectrum of **1** in CD_3OD (500MHz)

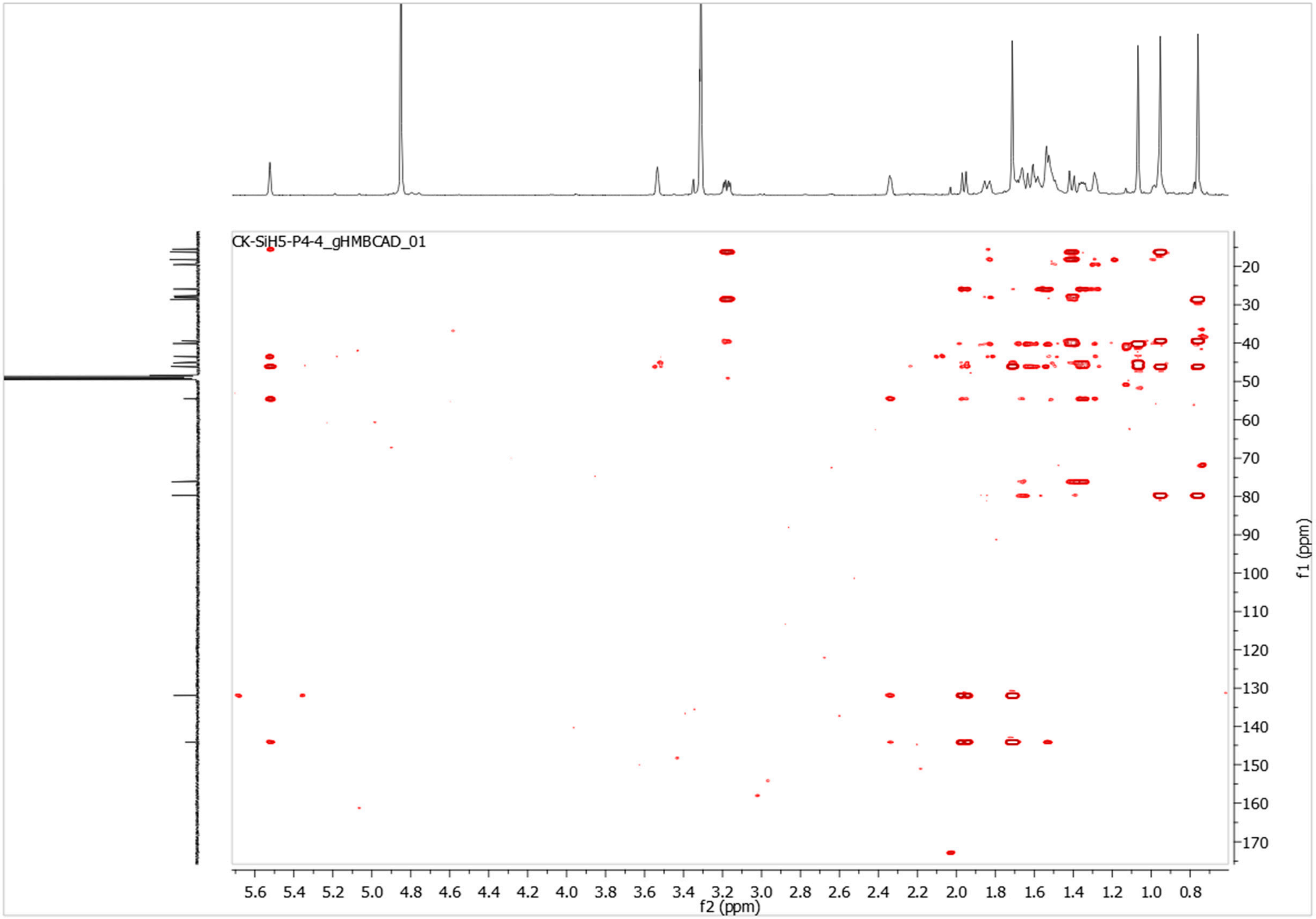

NOESY NMR spectrum of **1** in CD_3OD (500MHz)

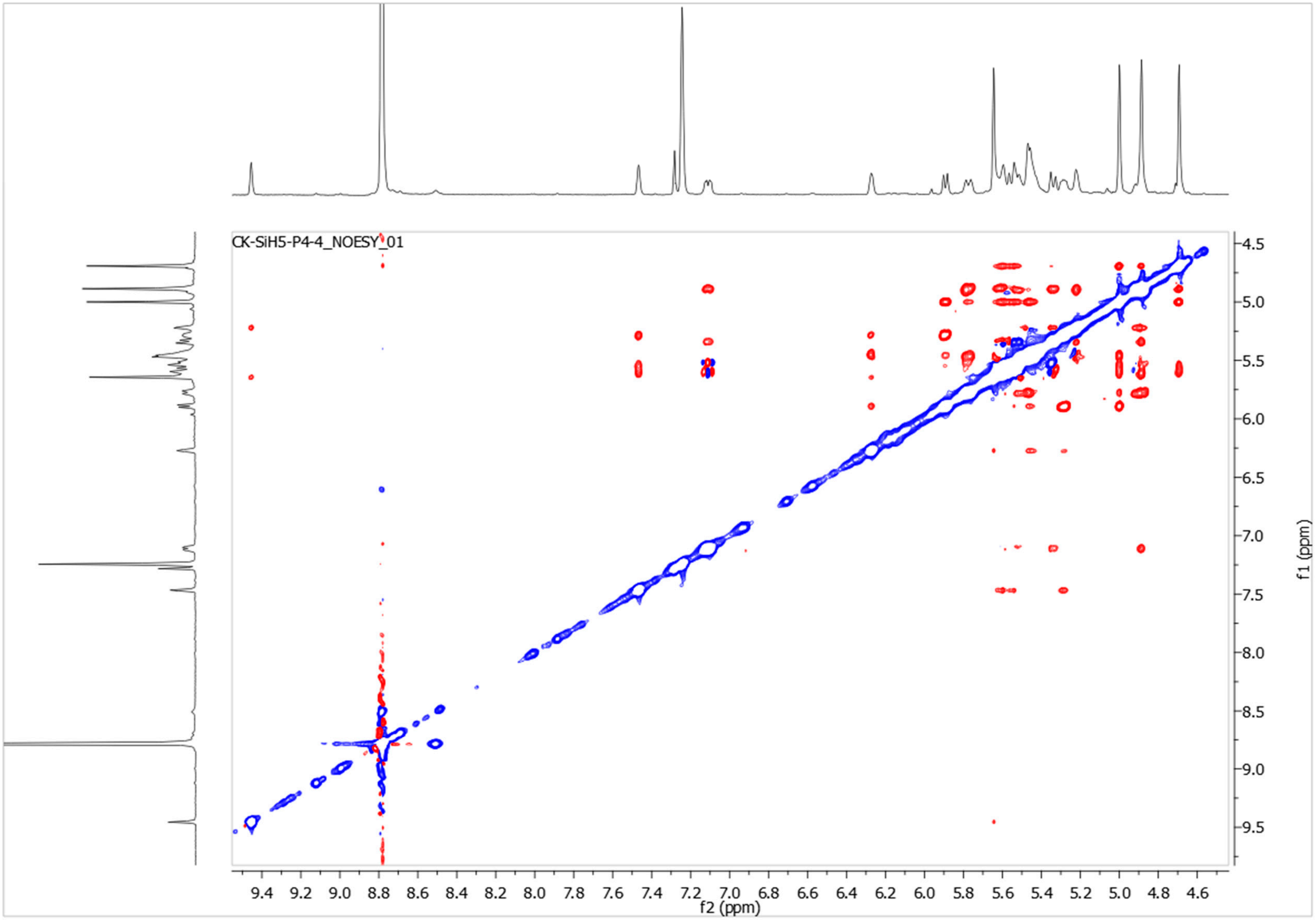

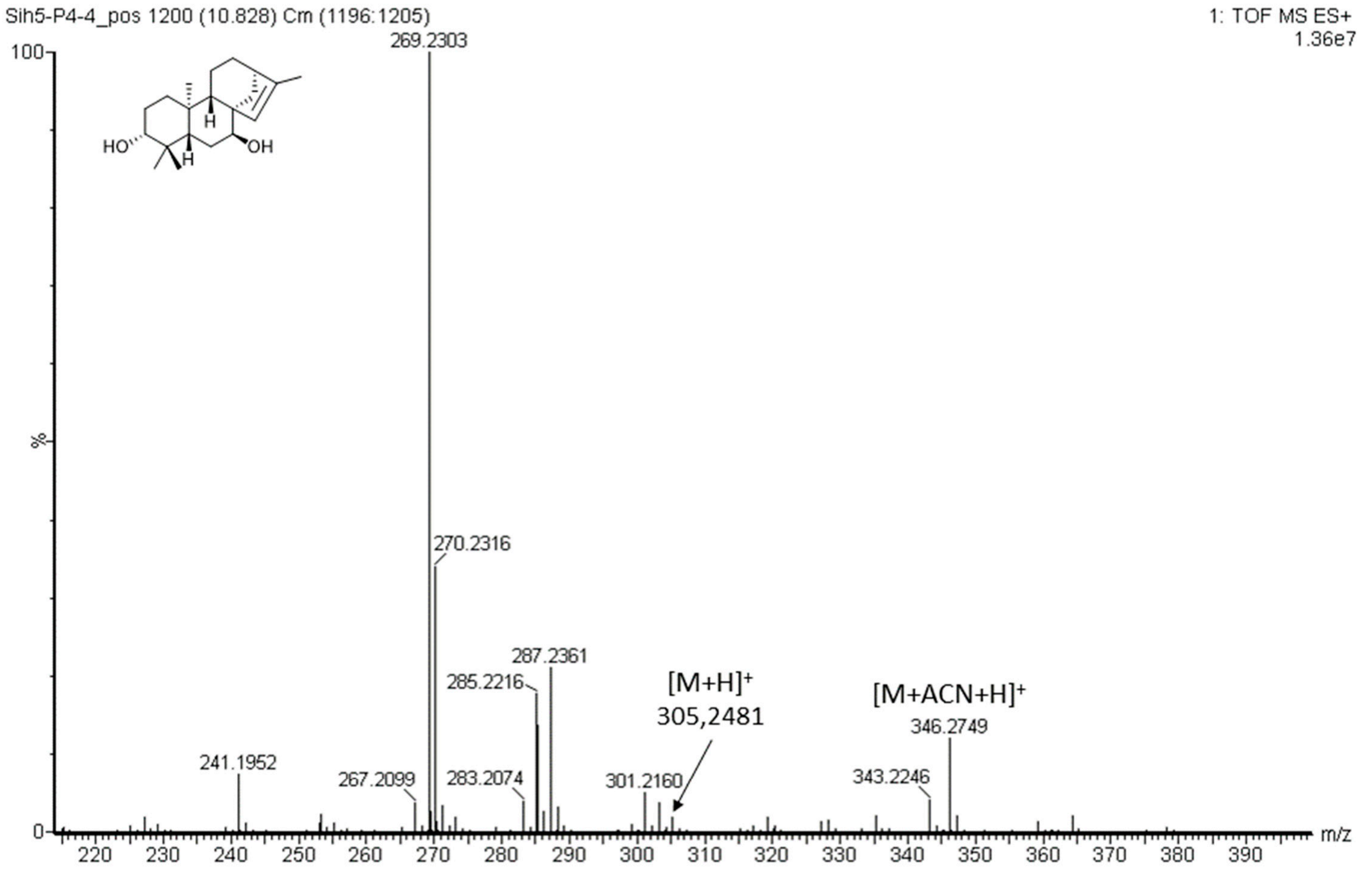

Sih5-P4-4_pos 1200 (10.828) Cm (1196:1205)
1: TOF MS ES+
1.36e7
100
%
0
HO
H
H
OH
269.2303
270.2316
287.2361
285.2216
[M+H]+
305,2481
[M+ACN+H]+
346.2749
241.1952
267.2099
283.2074
301.2160
343.2246
220
230
240
250
260
270
280
290
300
310
320
330
340
350
360
370
380
390
m/z

^{1}H NMR spectrum of **2** in CD_3OD (500MHz)

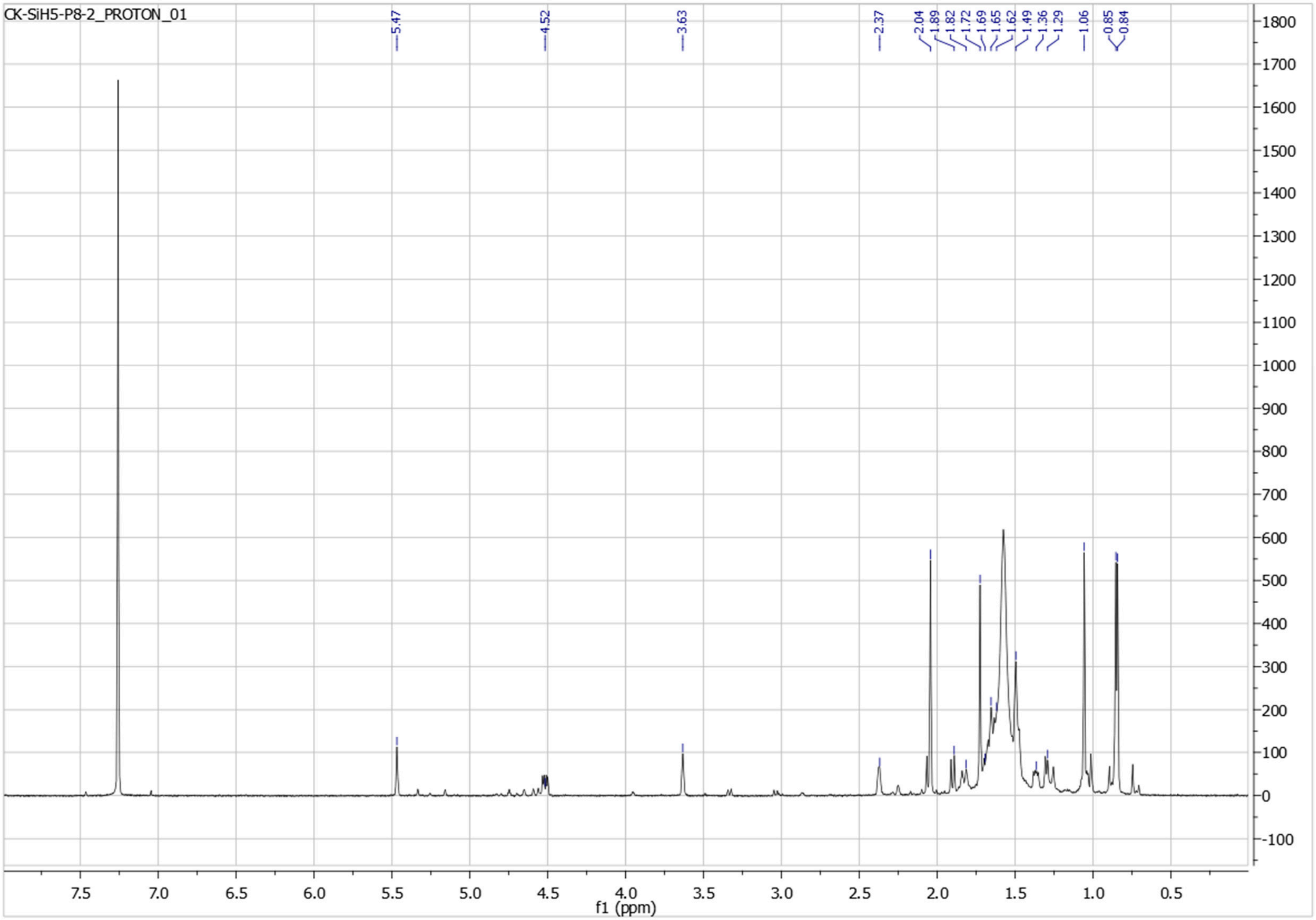

^{13}C NMR spectrum of **2** in CD_3OD (125MHz)

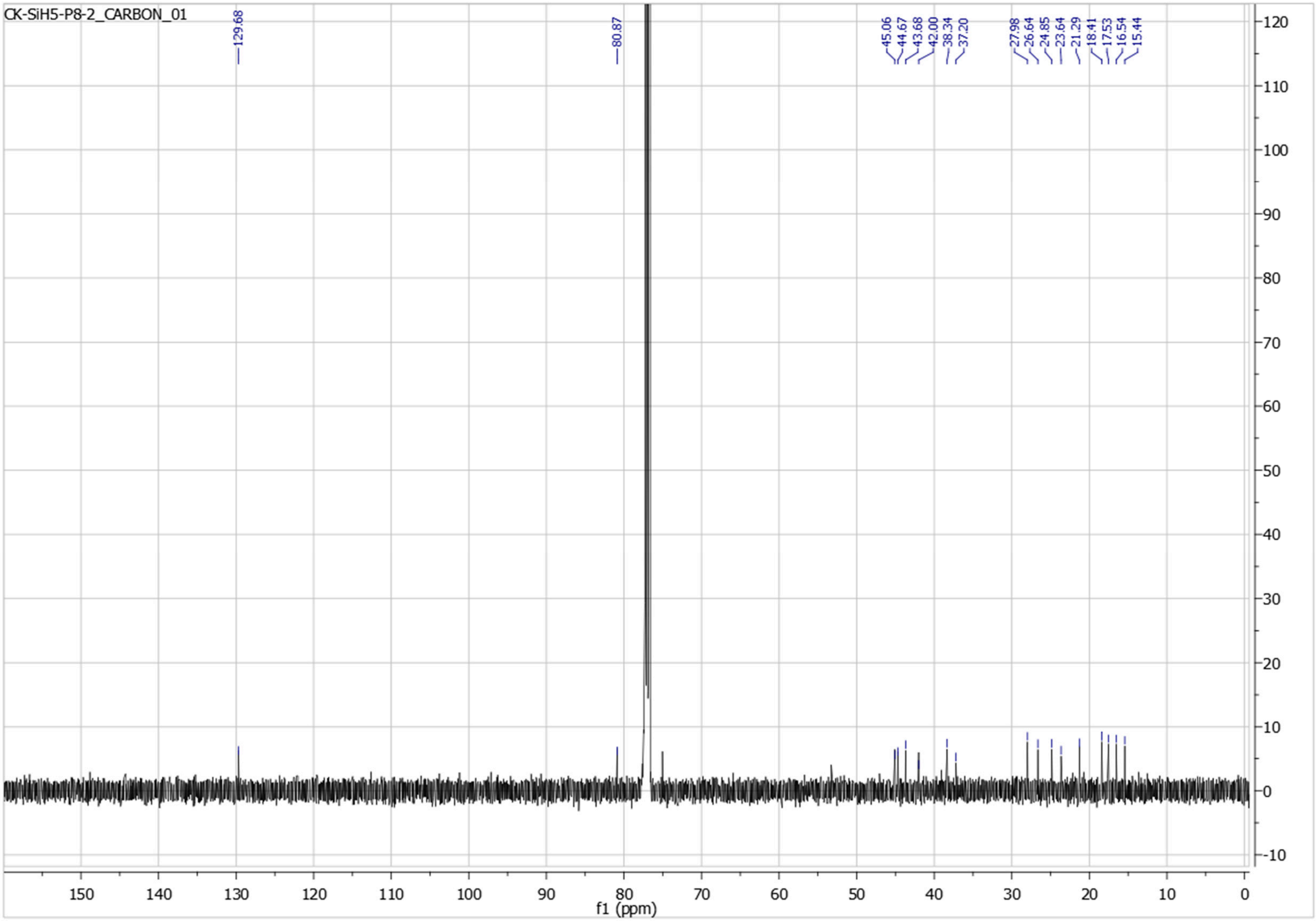

COSY NMR spectrum of **2** in CD_3OD (500MHz)

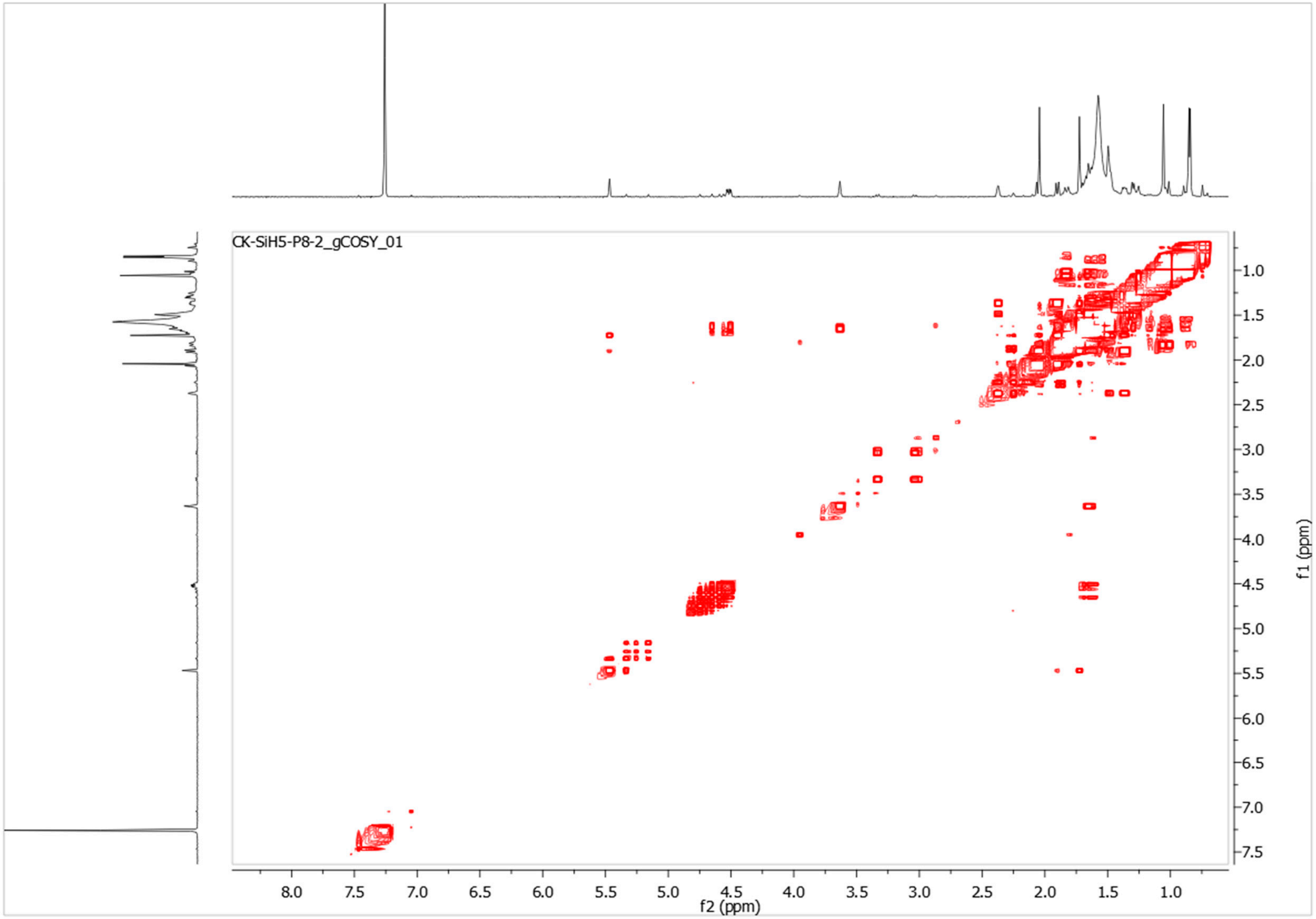

HMBC NMR spectrum of **2** in CD_3OD (500MHz)

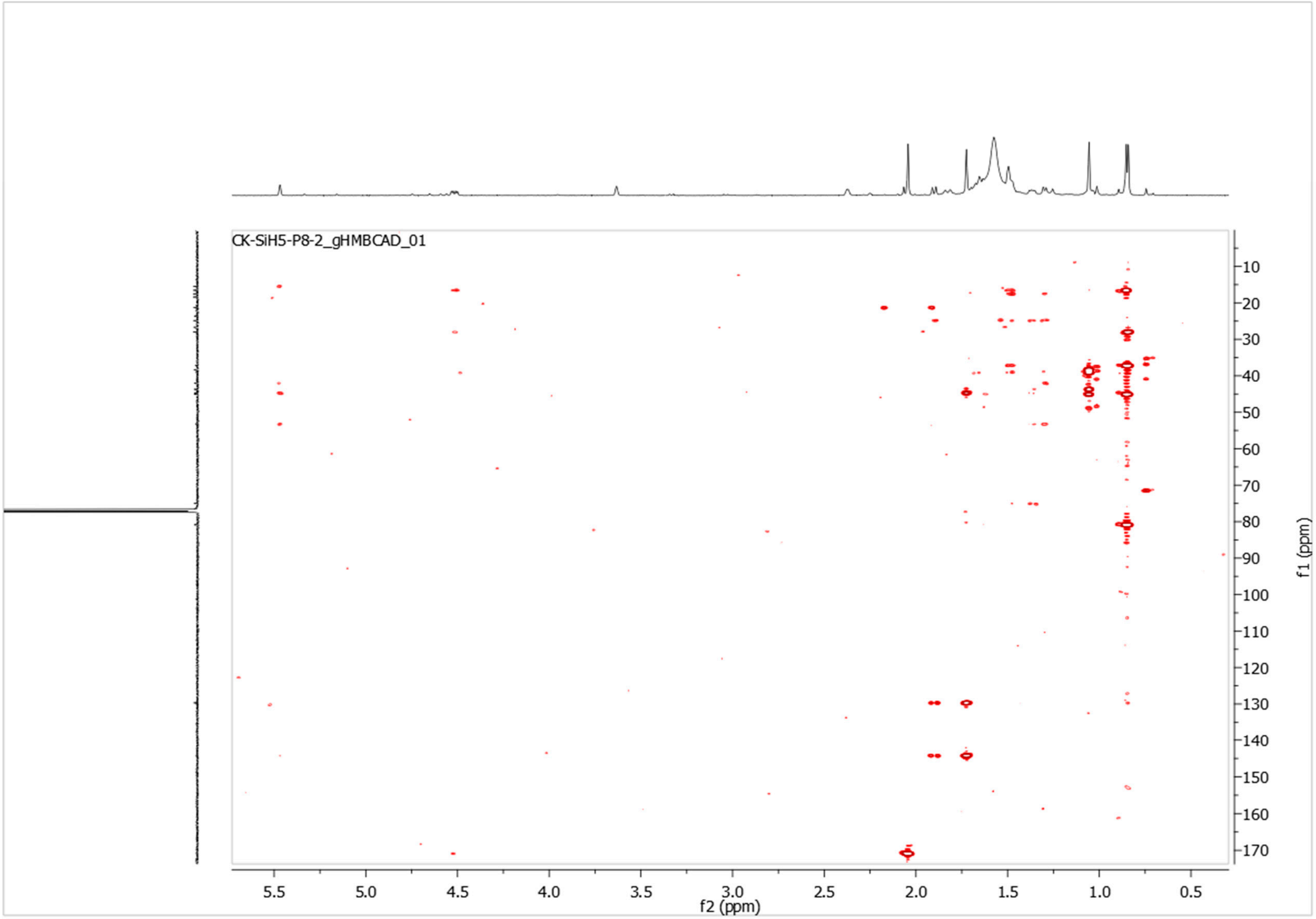

HSQC NMR spectrum of **2** in CD_3OD (500MHz)

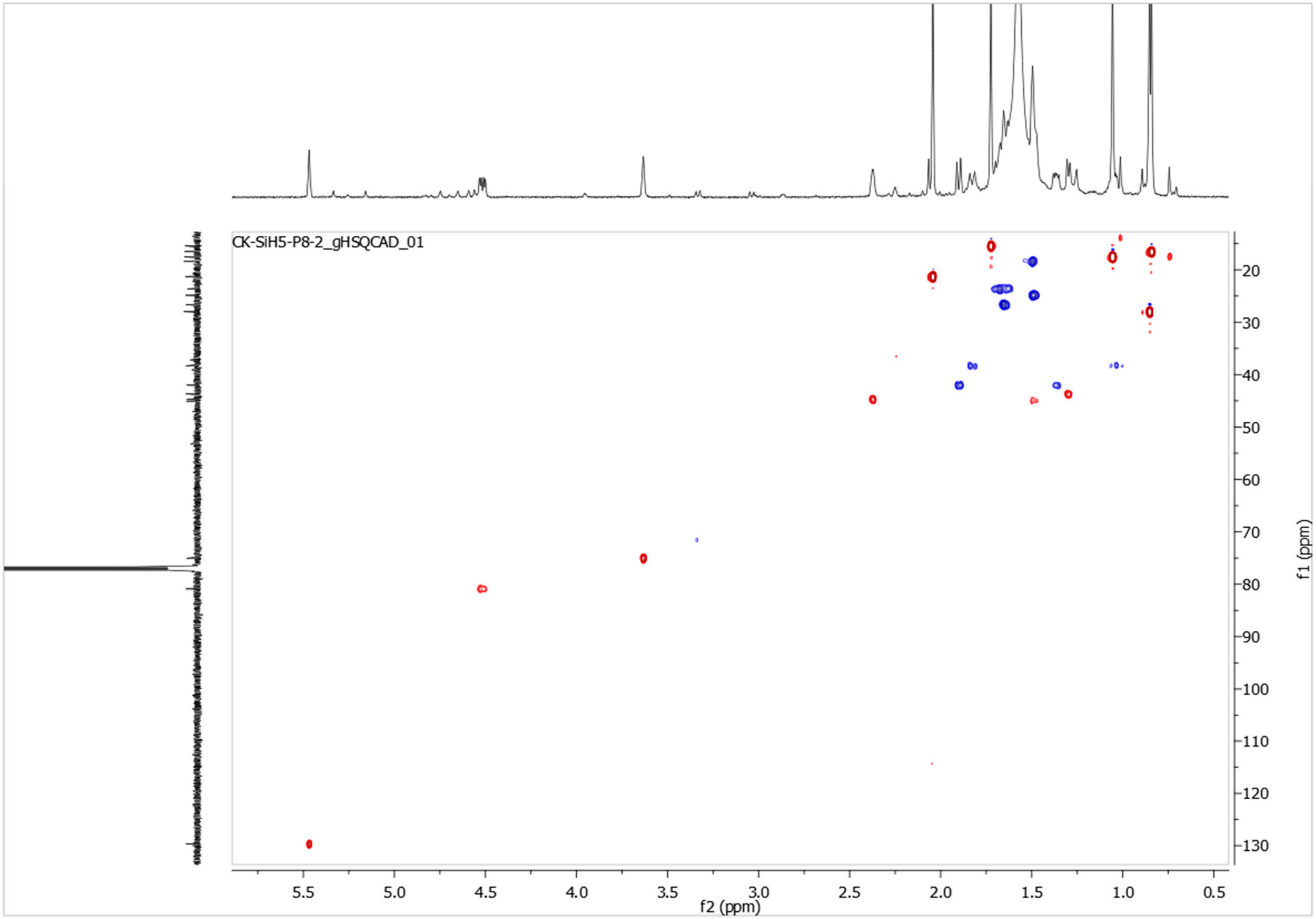

NOESY NMR spectrum of **2** in CD_3OD (500MHz)

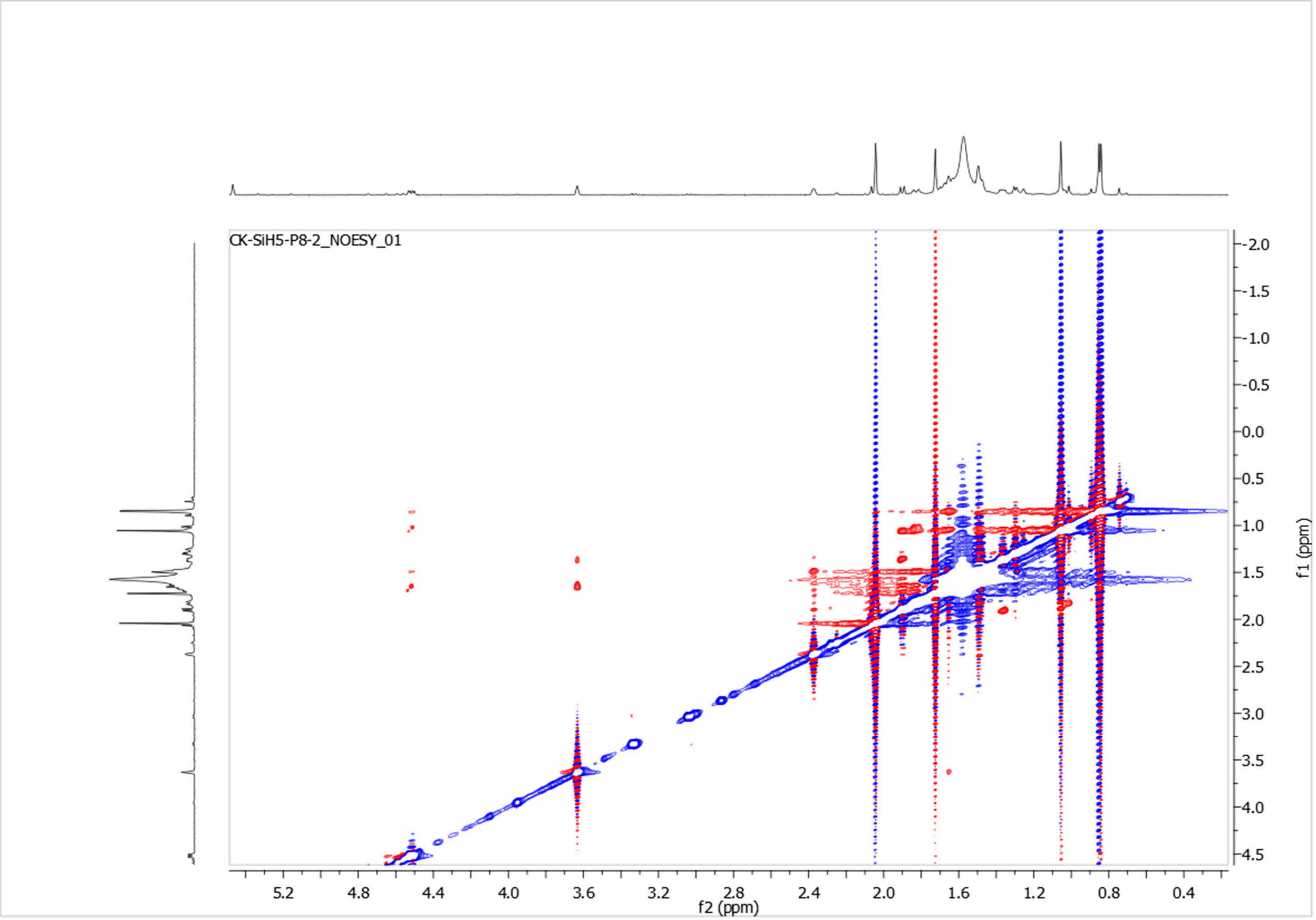

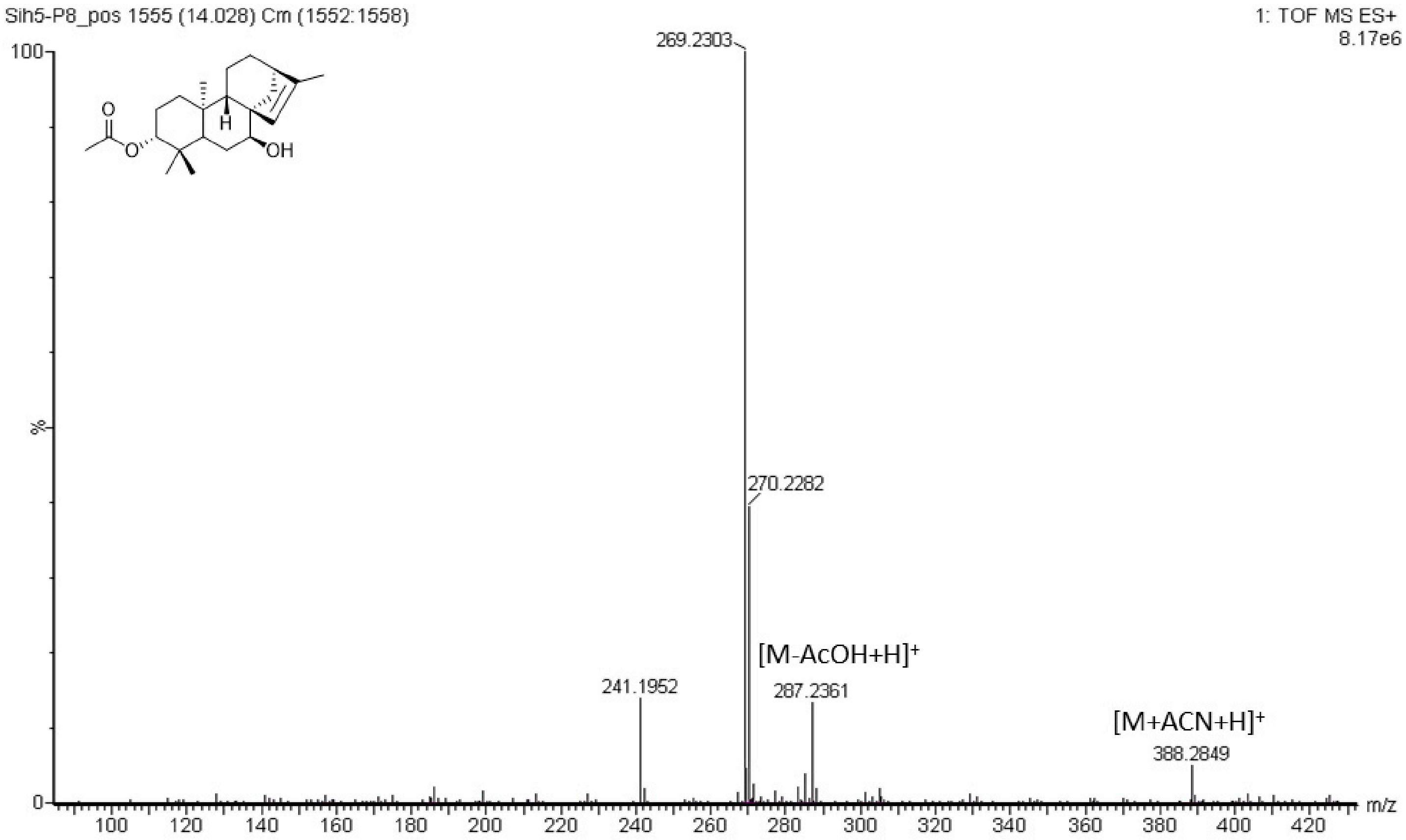

Sih5-P8_pos 1555 (14.028) Cm (1552:1558)
1: TOF MS ES+
8.17e6
269.2303
270.2282
[M-AcOH+H]+
241.1952
287.2361
[M+ACN+H]+
388.2849
%
m/z
O
OH
H

^1H NMR spectrum of **3** in CD_3OD (500MHz)

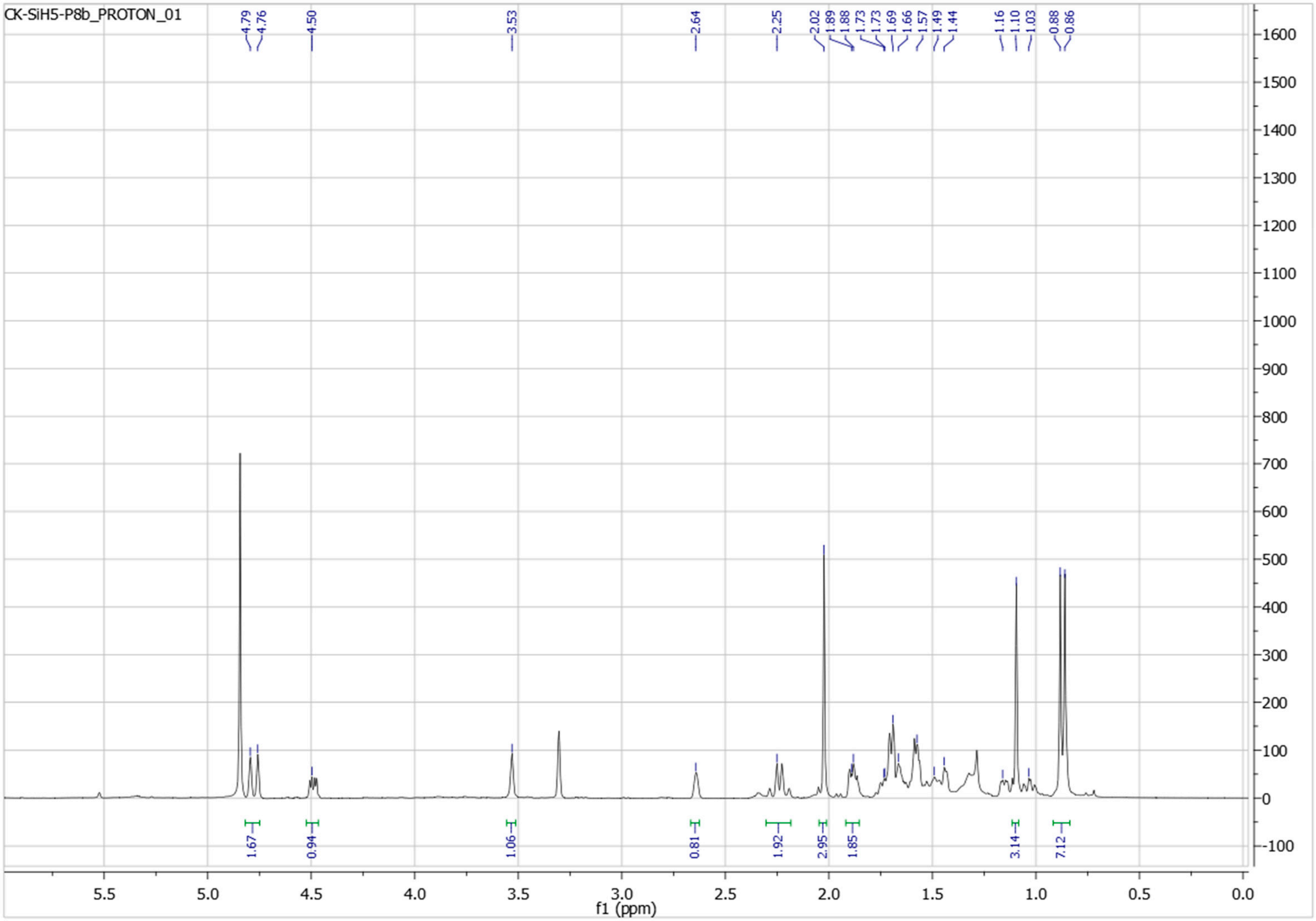

^{13}C NMR spectrum of **3** in CD_3OD (125MHz)

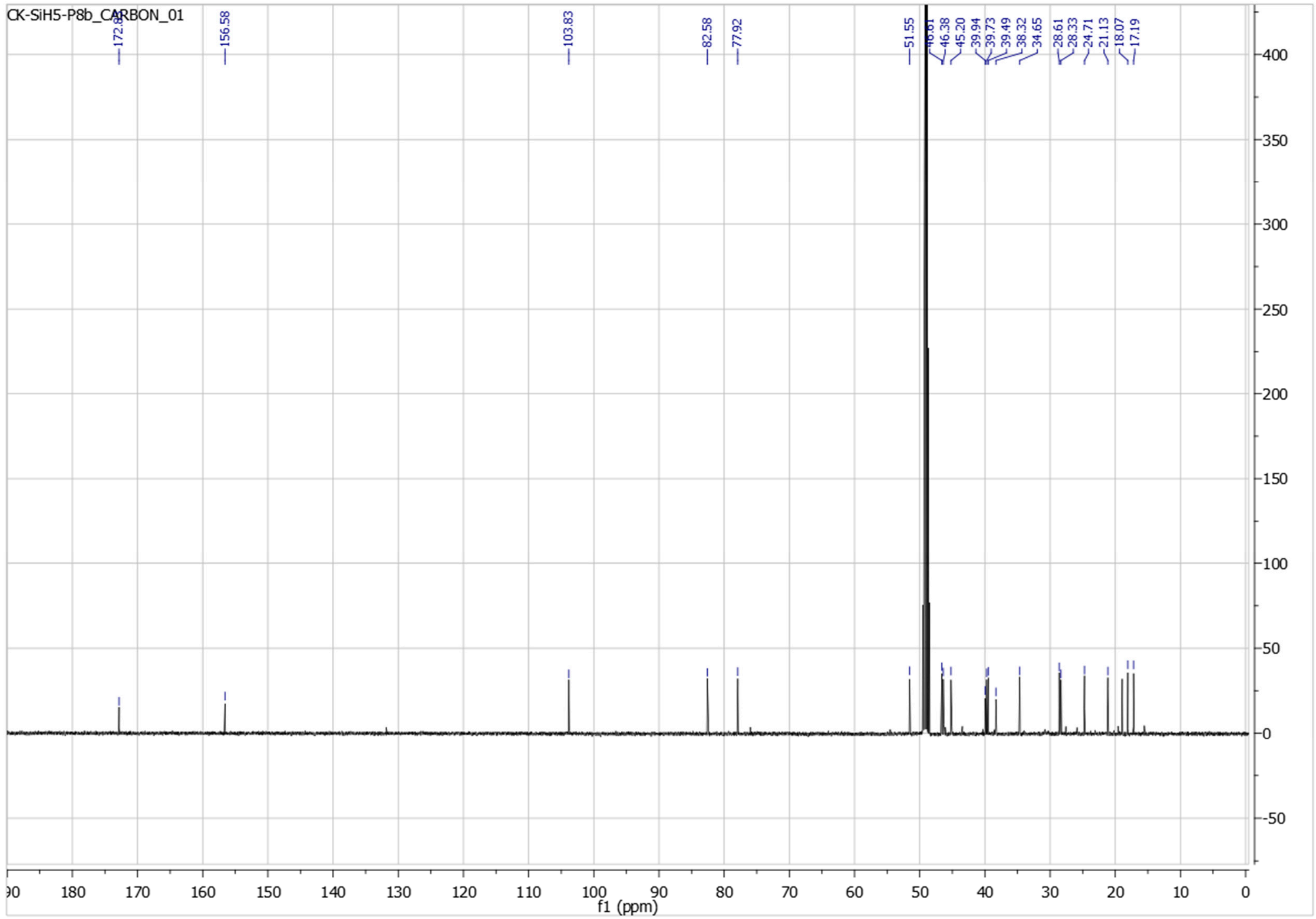

COSY NMR spectrum of **3** in CD_3OD (500MHz)

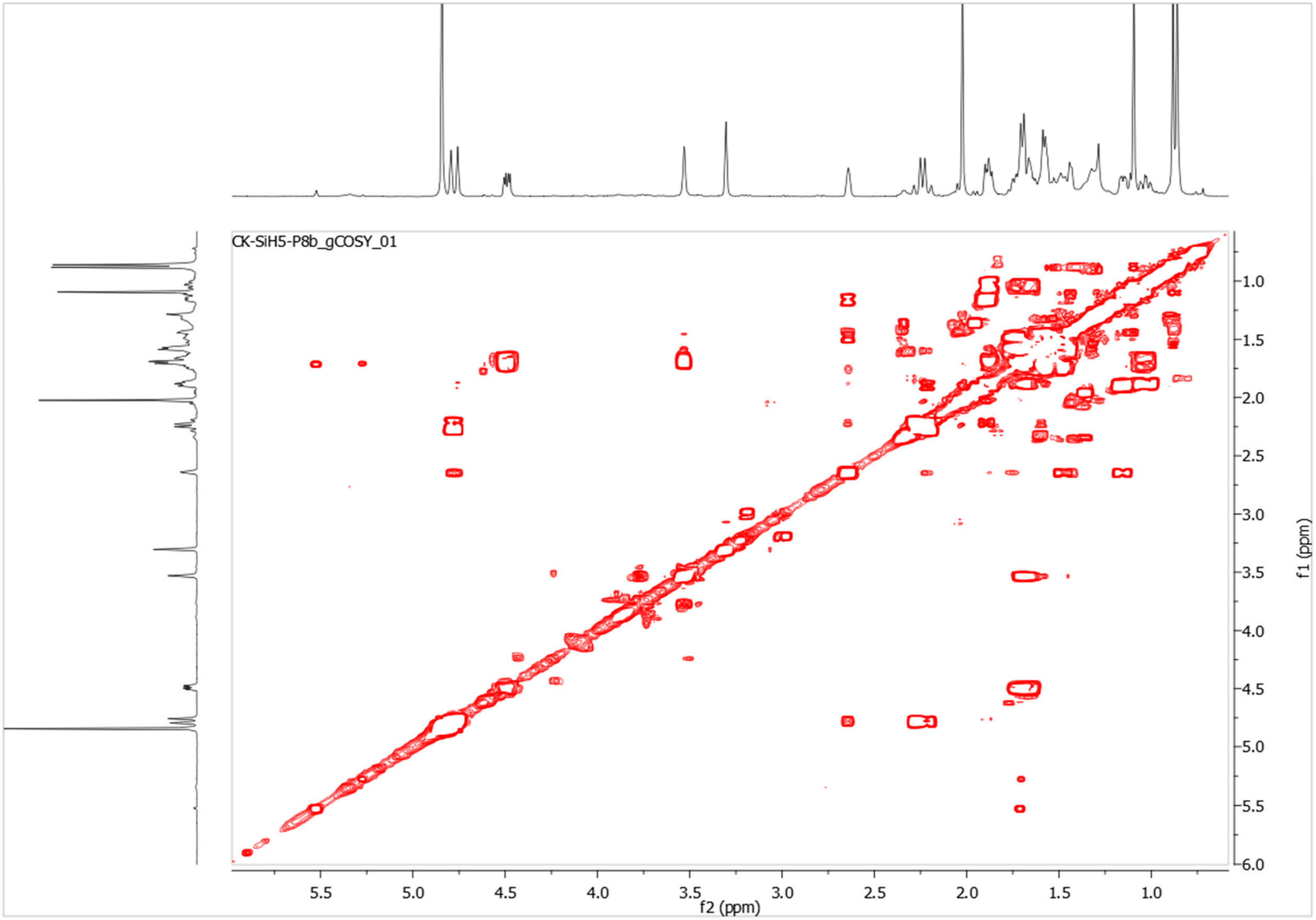

HSQC NMR spectrum of **3** in CD_3OD (500MHz)

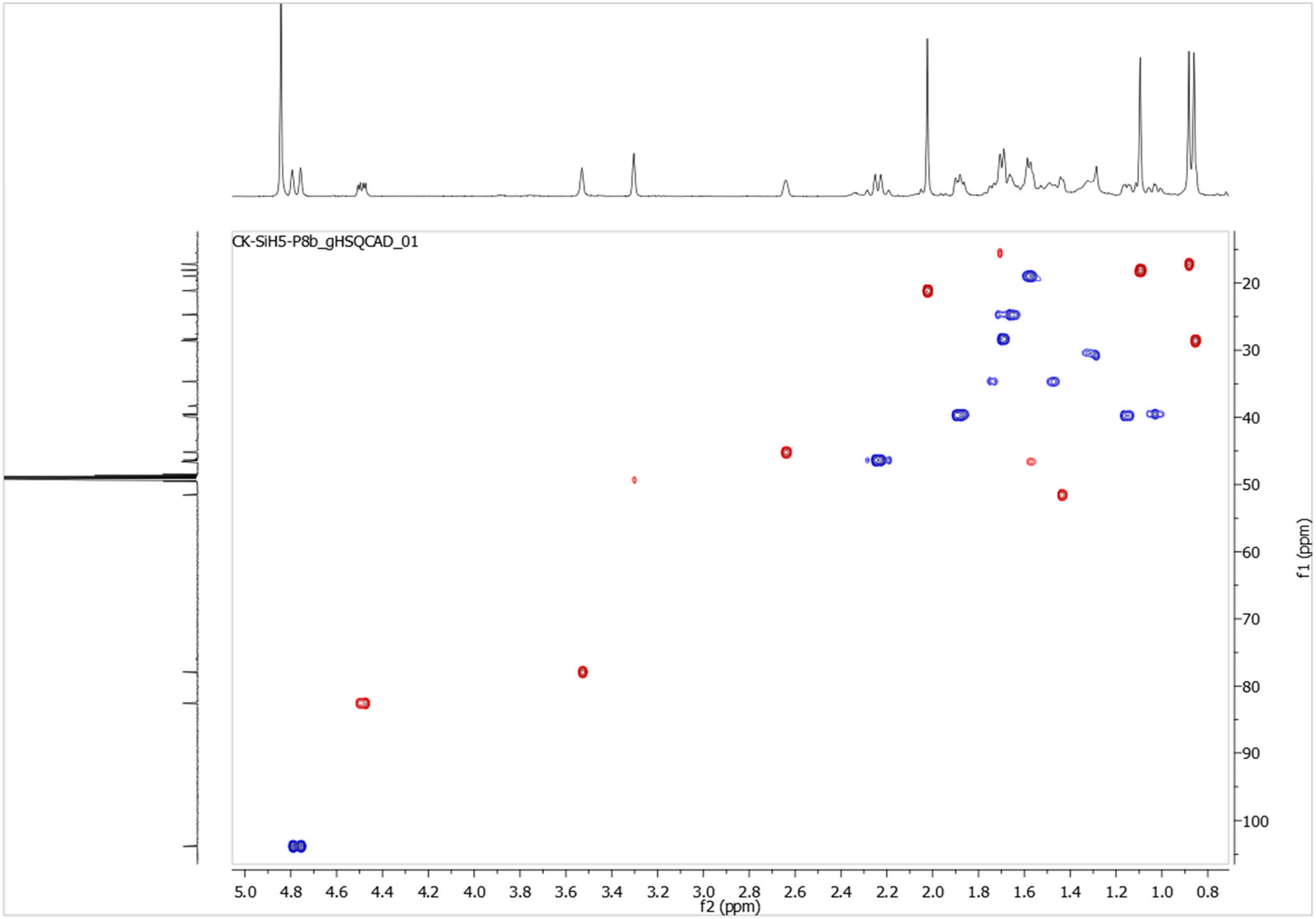

HMBC NMR spectrum of **3** in CD_3OD (500MHz)

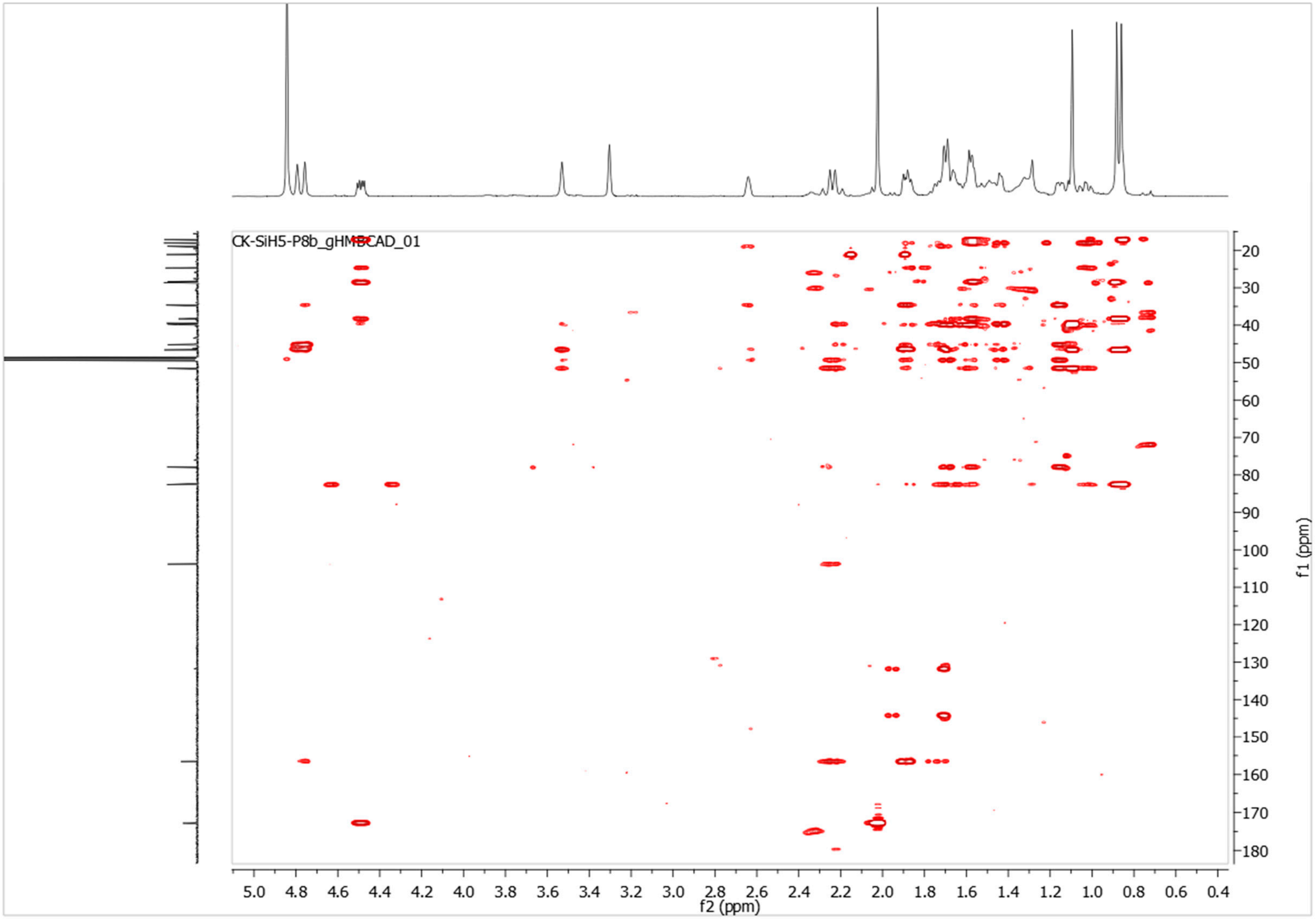

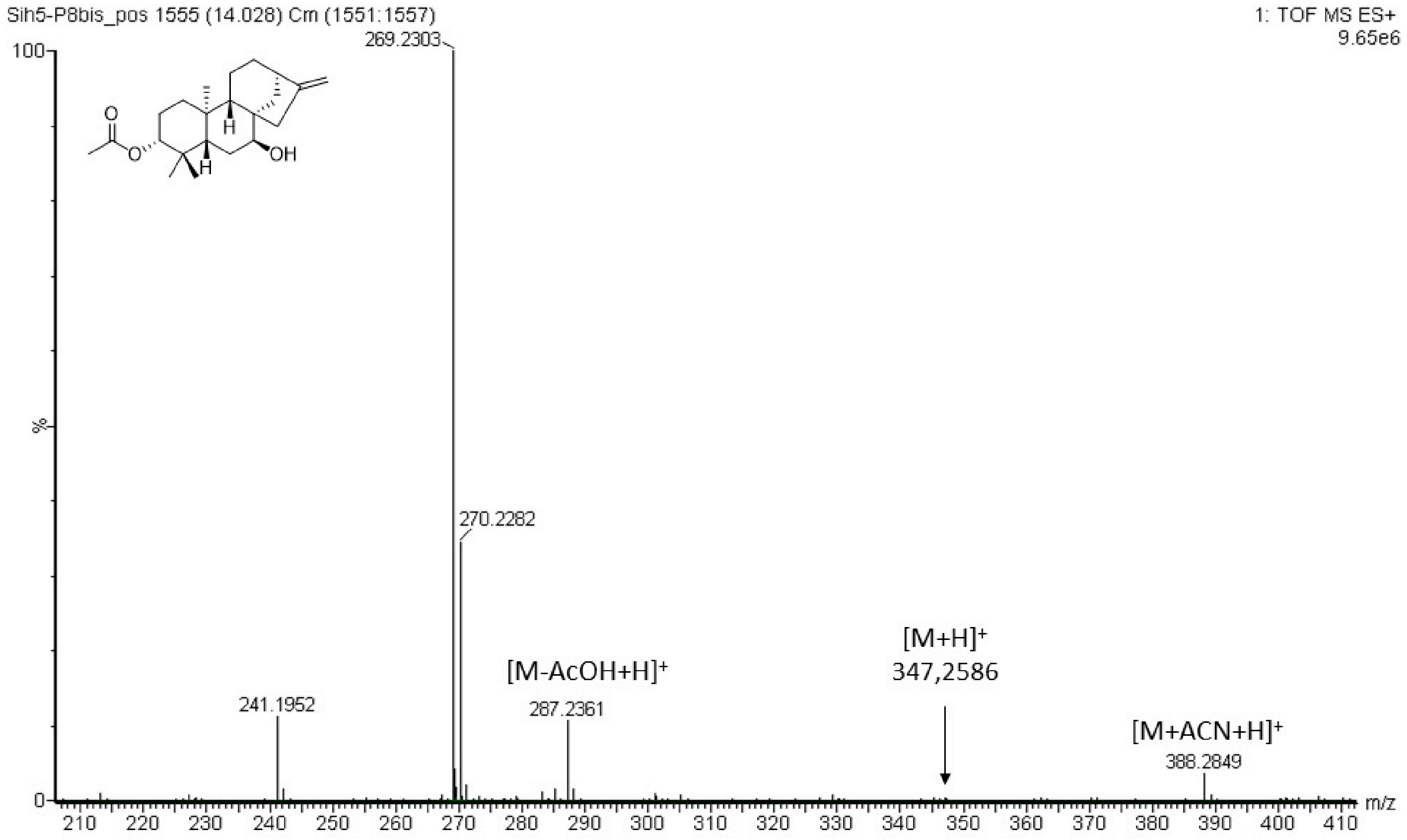

Sih5-P8bis_pos 1555 (14.028) Cm (1551:1557)
1: TOF MS ES+
9.65e6
100
%
0
269.2303
270.2282
241.1952
[M-AcOH+H]+
287.2361
[M+H]+
347,2586
[M+ACN+H]+
388.2849
OH
O
H
210 220 230 240 250 260 270 280 290 300 310 320 330 340 350 360 370 380 390 400 410
m/z

ECD spectra of **1-3**

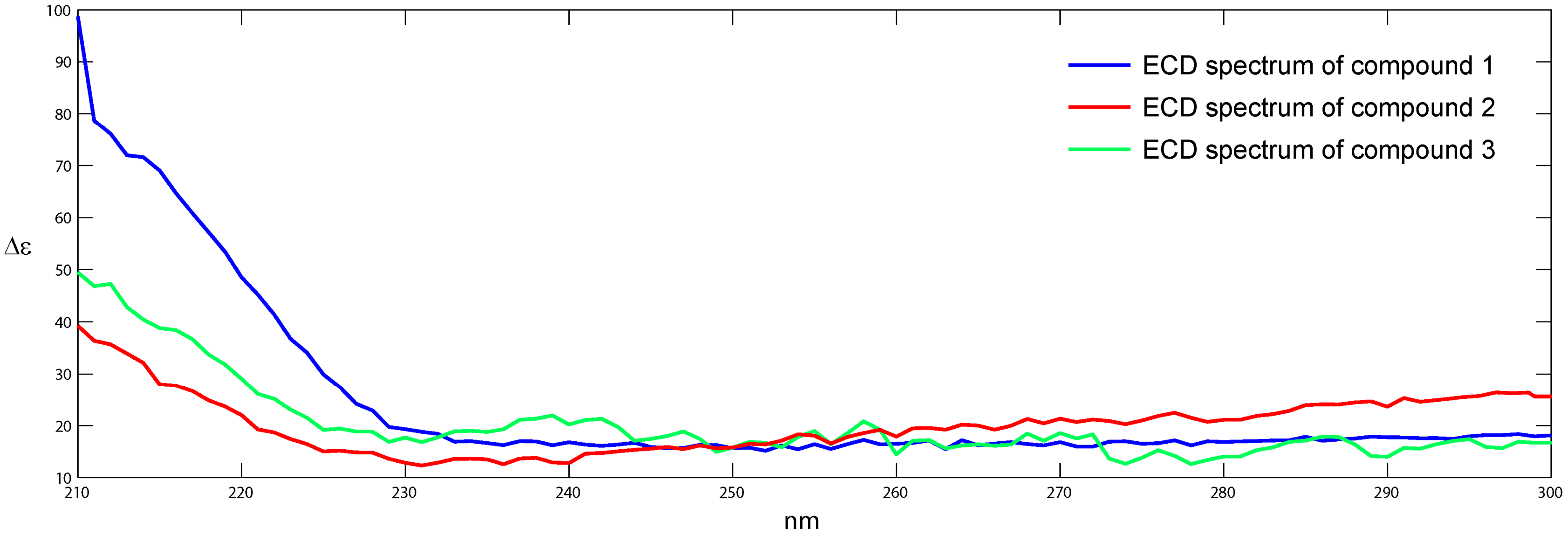

^1H NMR spectrum of **4** in CD_3OD (500MHz)

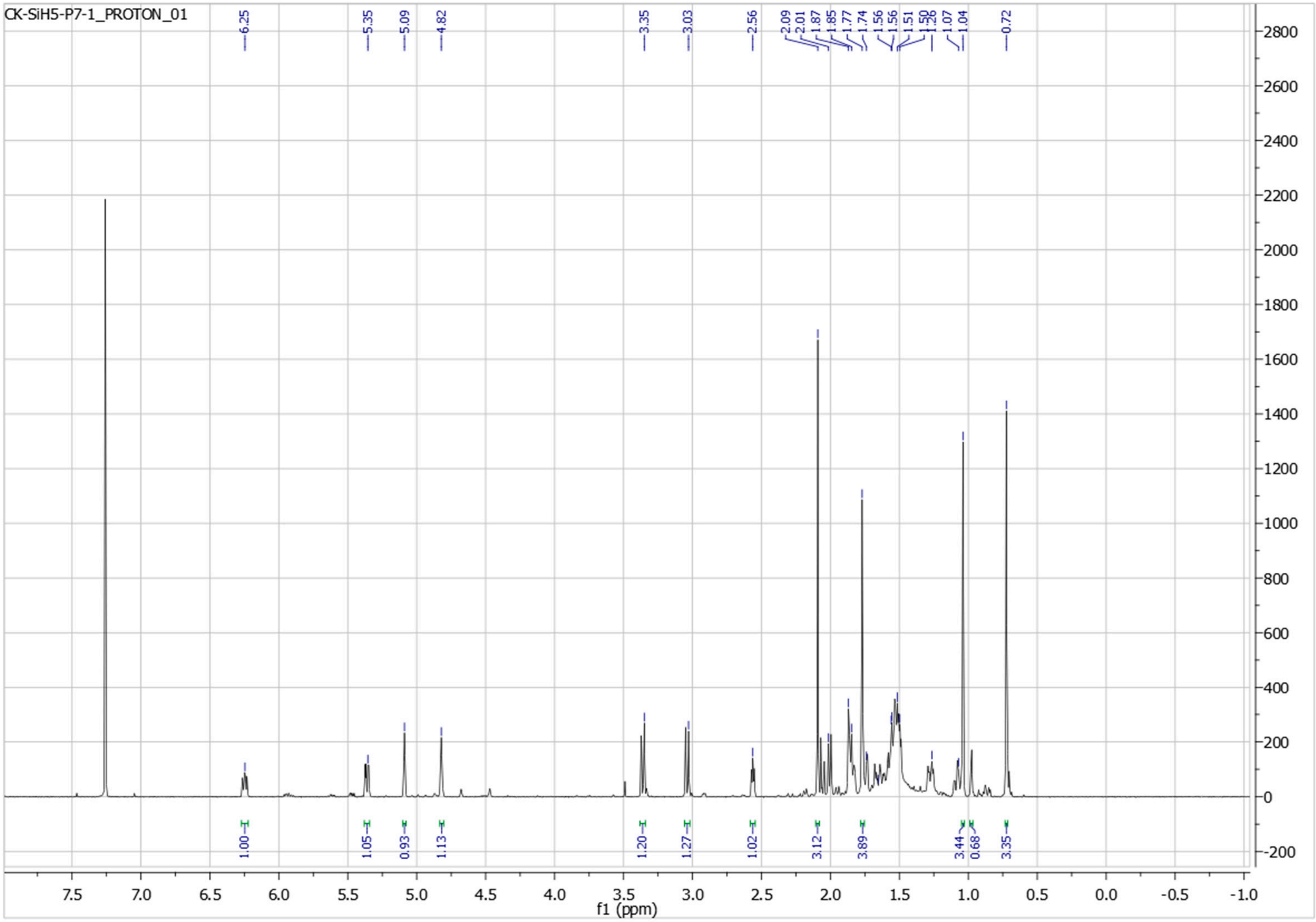

^{13}C NMR spectrum of **4** in CD_3OD (125MHz)

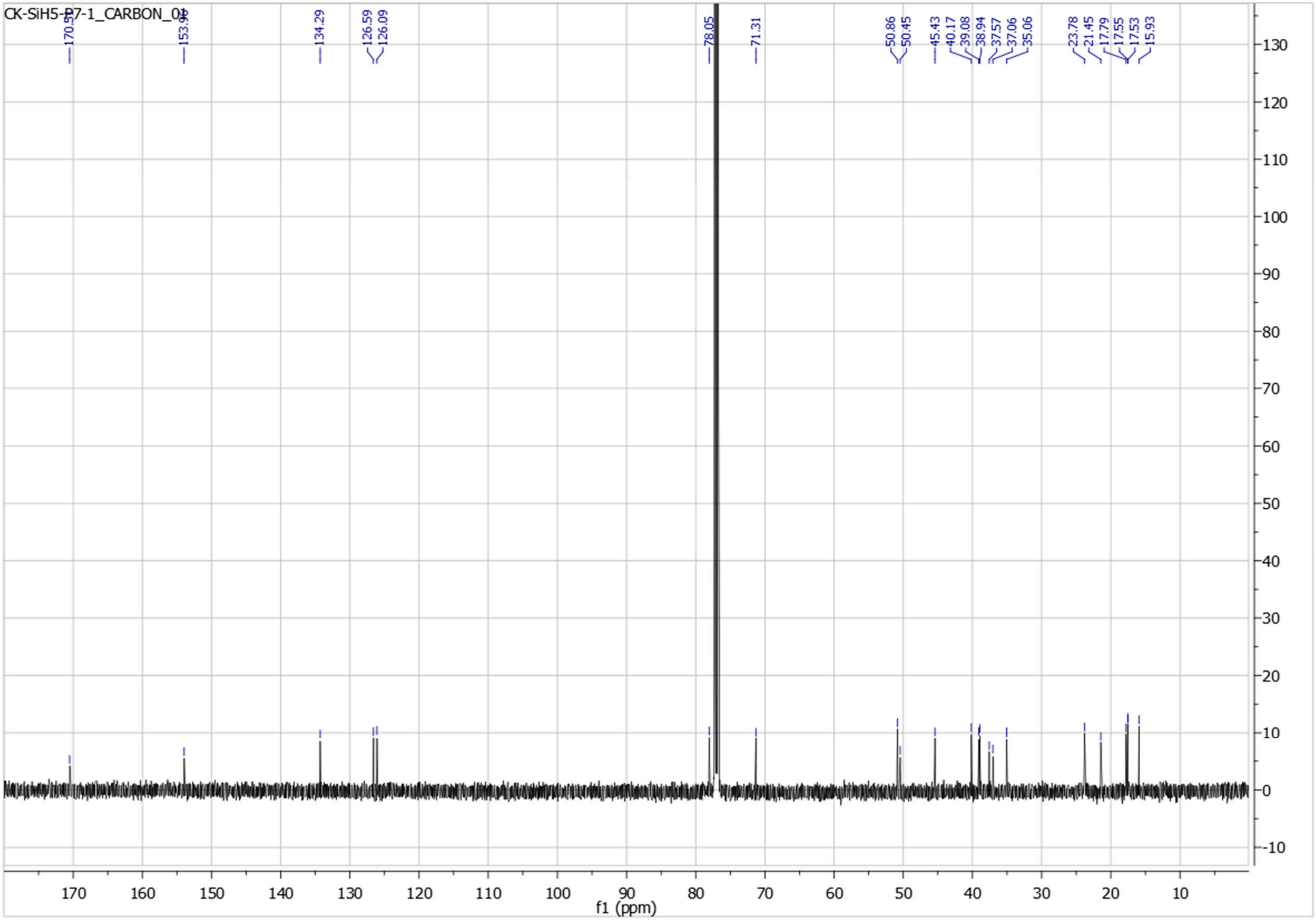

COSY NMR spectrum of **4** in CD_3OD (500MHz)

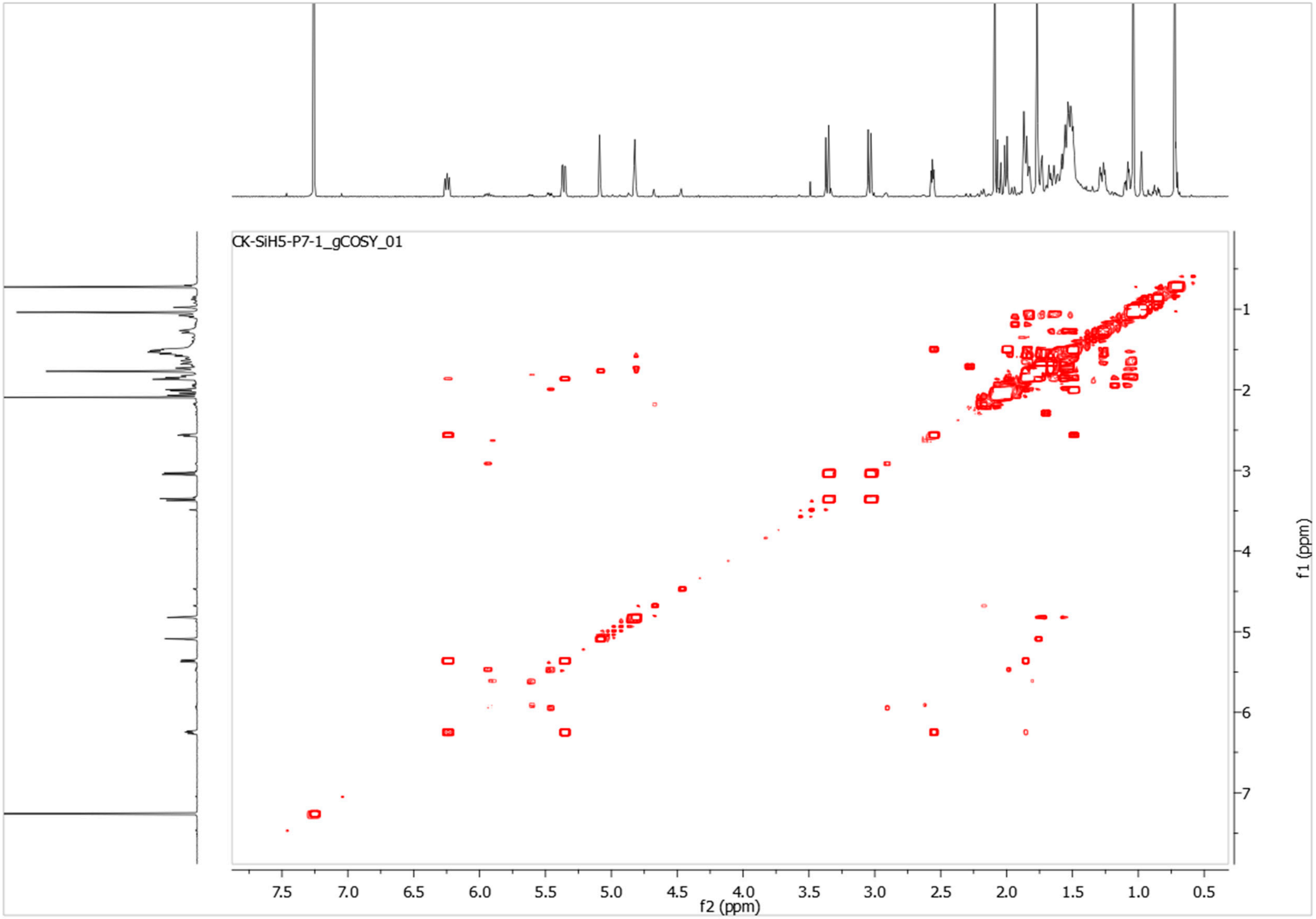

HSQC NMR spectrum of **4** in CD_3OD (500MHz)

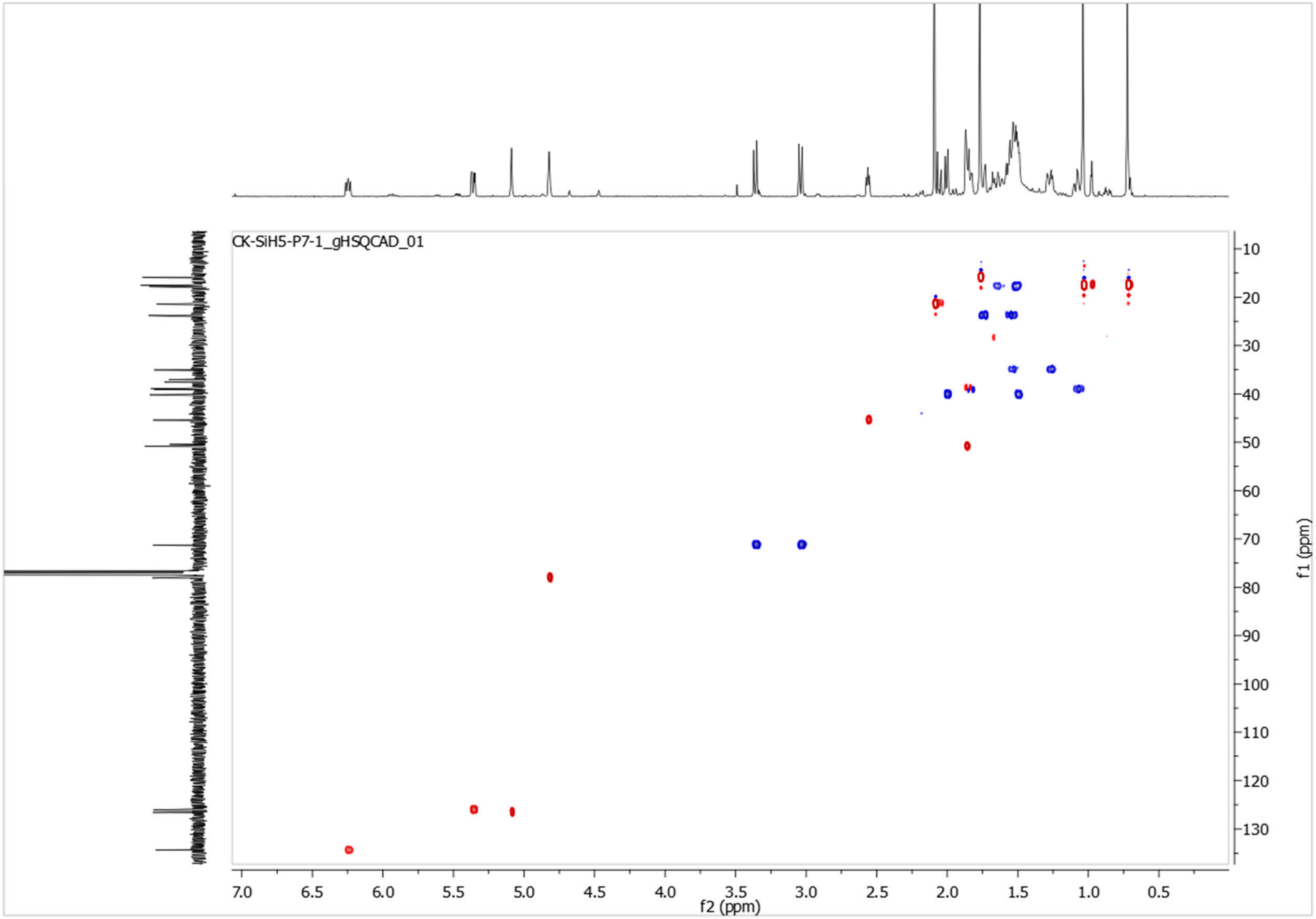

HMBC NMR spectrum of **4** in CD_3OD (500MHz)

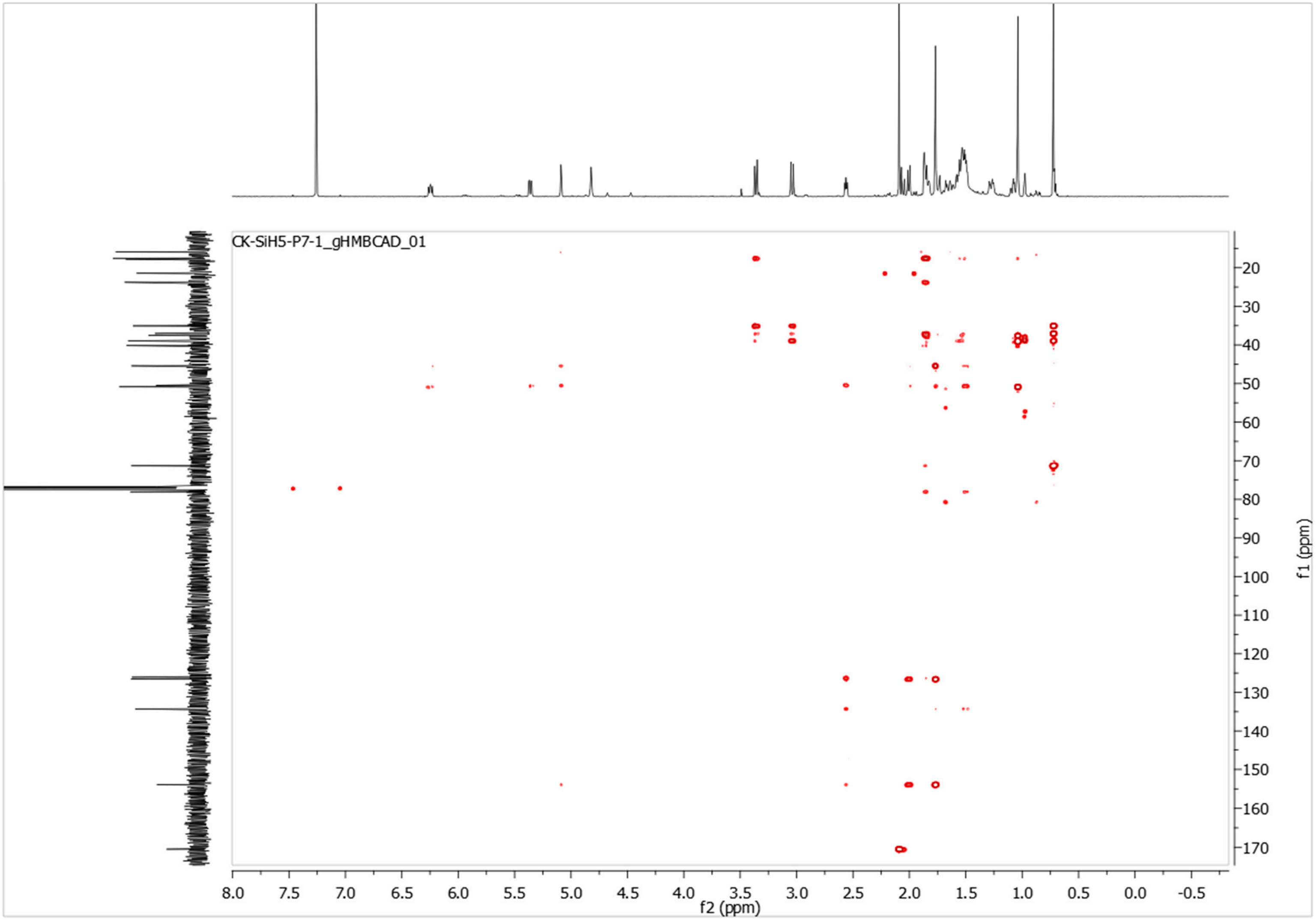

NOESY NMR spectrum of **4** in CD_3OD (500MHz)

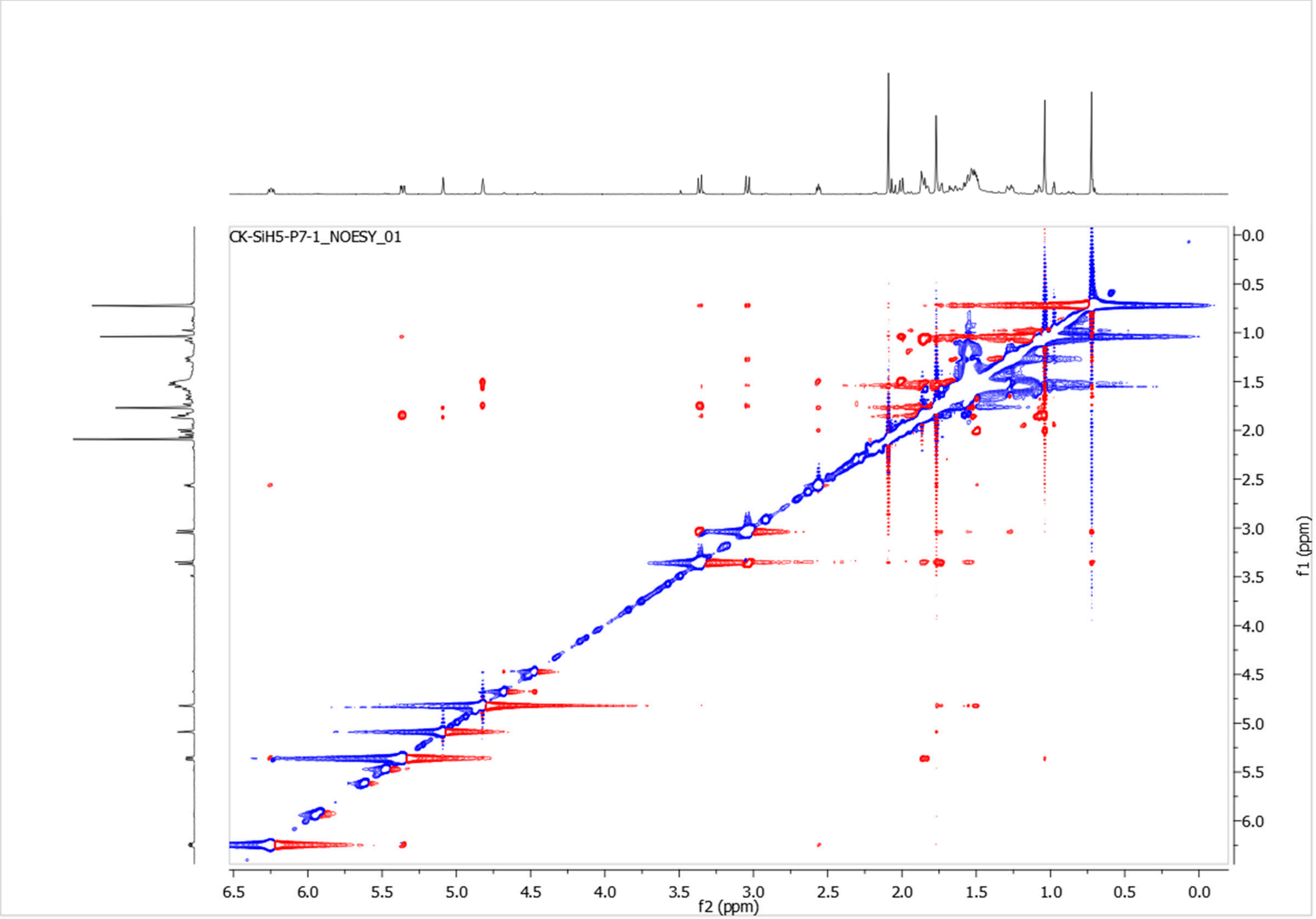

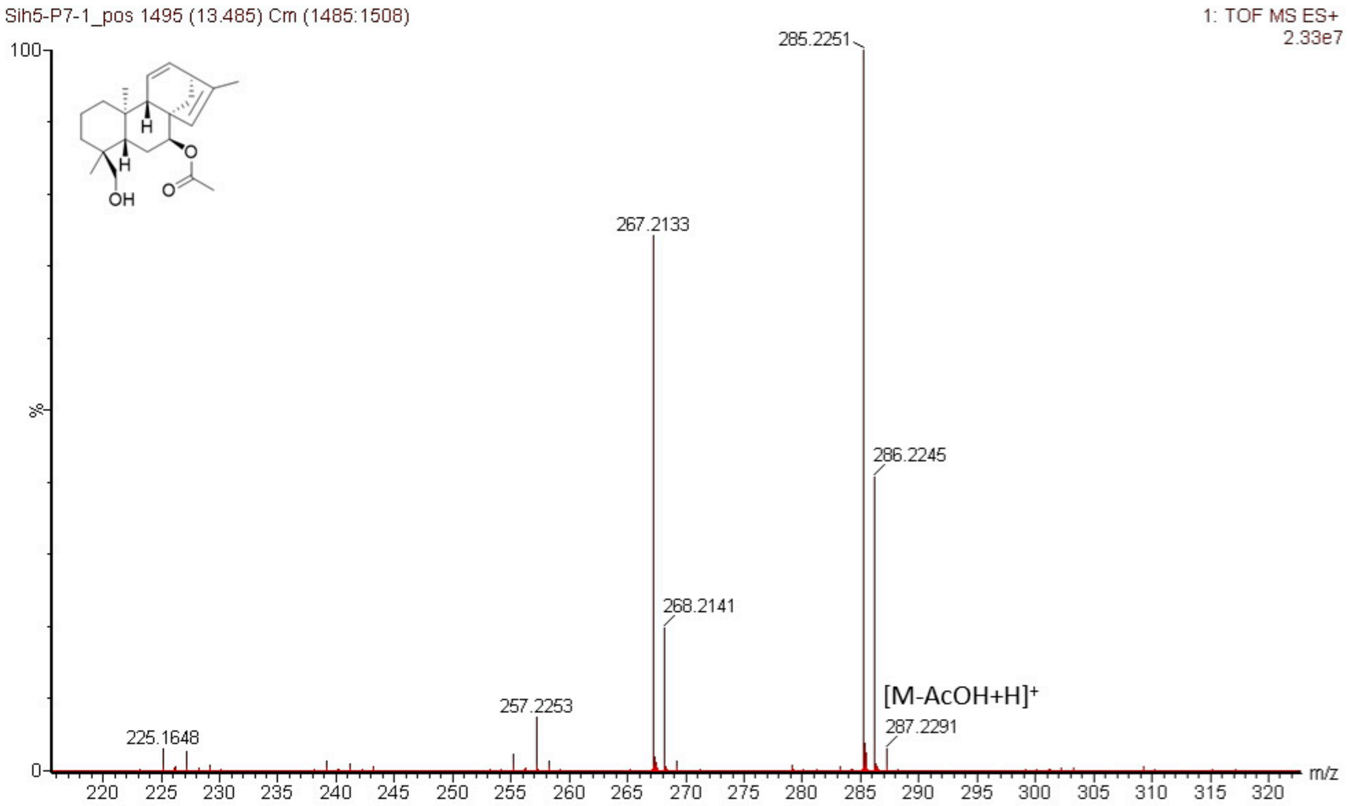

Sih5-P7-1_pos 1495 (13.485) Cm (1485:1508)
1: TOF MS ES+
2.33e7
100
%
0
OH
O
O
H
H
285.2251
267.2133
286.2245
268.2141
257.2253
225.1648
287.2291
[M-AcOH+H]+
220
225
230
235
240
245
250
255
260
265
270
275
280
285
290
295
300
305
310
315
320
m/z
